# *Pint *lincRNA connects the p53 pathway with epigenetic silencing by the Polycomb repressive complex 2

**DOI:** 10.1186/gb-2013-14-9-r104

**Published:** 2013-09-26

**Authors:** Oskar Marín-Béjar, Francesco P Marchese, Alejandro Athie, Yolanda Sánchez, Jovanna González, Victor Segura, Lulu Huang, Isabel Moreno, Alfons Navarro, Mariano Monzó, Jesús García-Foncillas, John L Rinn, Shuling Guo, Maite Huarte

**Affiliations:** 1 Center for Applied Medical Research, University of Navarra, 55 Pio XII Avenue., 31008 Pamplona, Spain; 2 Department of Antisense Drug Discovery, Isis Pharmaceuticals, 2855 Gazelle Court., Carlsbad, CA 92008, USA; 3 Department of Medical Oncology, Hospital Municipal de Badalona, 9-13 Via Augusta Street, 08911 Badalona, Spain; 4 Molecular Oncology and Embryology Laboratory, Human Anatomy Unit, University of Barcelona Medical School, IDIBAPS, 143 Casanova Street, 080360 Barcelona, Spain; 5 Department of Oncology, Translational Oncology Division, Health Research Institute Fundación Jiménez Díaz University Hospital, Autonomous University of Madrid, 2 Reyes Catolicos Avenue, 28040 Madrid, Spain; 6 Stem Cell and Regenerative Biology, Harvard University, 7 Divinity Avenue, Cambridge, MA 02138, USA

**Keywords:** lincRNA, non-coding RNA, p53, gene regulation, Polycomb repressive complex 2

## Abstract

**Background:**

The p53 transcription factor is located at the core of a complex wiring of signaling pathways that are critical for the preservation of cellular homeostasis. Only recently it has become clear that p53 regulates the expression of several long intergenic noncoding RNAs (lincRNAs). However, relatively little is known about the role that lincRNAs play in this pathway.

**Results:**

Here we characterize a lincRNA named *Pint *(p53 induced noncoding transcript). We show that *Pint *is a ubiquitously expressed lincRNA that is finely regulated by p53. In mouse cells, *Pint *promotes cell proliferation and survival by regulating the expression of genes of the TGF-β, MAPK and p53 pathways. *Pint *is a nuclear lincRNA that directly interacts with the Polycomb repressive complex 2 (PRC2), and is required for PRC2 targeting of specific genes for H3K27 tri-methylation and repression. Furthermore, *Pint *functional activity is highly dependent on PRC2 expression. We have also identified *Pint *human ortholog (*PINT*), which presents suggestive analogies with the murine lincRNA. *PINT *is similarly regulated by p53, and its expression significantly correlates with the same cellular pathways as the mouse ortholog, including the p53 pathway. Interestingly, *PINT *is downregulated in colon primary tumors, while its overexpression inhibits the proliferation of tumor cells, suggesting a possible role as tumor suppressor.

**Conclusions:**

Our results reveal a p53 autoregulatory negative mechanism where a lincRNA connects p53 activation with epigenetic silencing by PRC2. Additionally, we show analogies and differences between the murine and human orthologs, identifying a novel tumor suppressor candidate lincRNA.

## Background

How cells coordinate and integrate information to produce adequate gene-expression output is still an unsolved question with important implications for biology and health. Even the slightest perturbation of cellular networks can affect homeostasis and lead to cell transformation. Of these cellular networks, the p53 pathway is possibly the most relevant for preservation of cellular homeostasis. The transcription factor p53 is located at the core of a complex wiring of signaling pathways, and it has been proposed as the master regulator of cell fate. The importance of the tumor suppressing functions of p53 is shown by its high mutation frequency in cancers and by the highly tumorigenic phenotype of p53 null mice [1].

We and others have shown that long intergenic non-coding RNAs (lincRNAs) are part of the p53 transcriptional network [2-4]. LincRNAs are intergenic transcripts longer than 200 nucleotides that lack functional open reading frames (ORFs). Although thousands of lincRNAs exist, only a relatively small number have been studied in some depth, indicating that lincRNAs have roles in numerous physiological processes that involve gene regulation [5,6]. Many of these lincRNAs have been shown to act as molecular scaffolds that hold and guide chromatin complexes [7-9]. In particular, several lincRNAs have been found to be associated with the Polycomb repressive complex 2 (PRC2) in a number of biological contexts, modulating PRC2-specific targeting of genes [8,10,11]. PRC2 is composed of three core components: Suppressor of zeste 12 (Suz12), Embryonic Ectoderm Development (EED), and the H3K27 histone methyl transferase Enhancer of zeste homolog 2 (Ezh2). PRC2 represses gene expression by catalyzing H3K27 tri-methylation and modulating chromatin structure [12], and is closely linked with the aberrant proliferation of cancer cells. For instance, the Suz12 subunit is overexpressed in colon and breast cancers [13], and Ezh2 is upregulated in a number of tumors, including Hodgkin lymphoma, prostate cancer, and breast cancer [14,15]. Moreover, Ezh2 expression is associated with poor prognosis, and is an indication of the metastatic potential of a tumor [15,16]. Similarly, alterations in expression of lincRNAs in cancer have been reported, implicating lincRNAs as possible attractive therapeutic targets [17,18].

In a previous work. we used mouse cell lines combined with custom microarrays to monitor the differential expression of lincRNAs, and found that p53 specifically activated several lincRNAs. We characterized one of them, lincRNA-p21, which was found to function as a transcriptional repressor [3]. However, the contribution of lincRNAs to p53 biology and to cancer still remains largely unexplored.

Here, we expand this knowledge by characterizing *Pint*. We show that *Pint *is a ubiquitously expressed mouse lincRNA that is a direct p53 transcriptional target. *Pint *acts as a positive regulator of cell proliferation and survival, affecting the expression of hundreds of genes, including a fraction of the p53 transcriptional network. *PINT *interacts with PRC2 and is required for PRC2 targeting of specific genes for H3K27 tri-methylation and repression. We also show that the *PINT *human ortholog is similarly regulated by p53. Interestingly, whereas in normal tissue, *PINT *shows a significant inverse correlation with the p53 pathway, it is downregulated in colorectal cancer, and its enforced expression inhibits the proliferation of tumor cells. To our knowledge, the results presented here represent the first experimentally supported connection between the p53 pathway and Polycomb epigenetic regulation mediated by a lincRNA. Moreover, the data suggest that *PINT *may serve as a novel tumor suppressor.

## Results

### *Pint*, a long non-coding RNA transcriptionally regulated by p53

**Figure 1:**
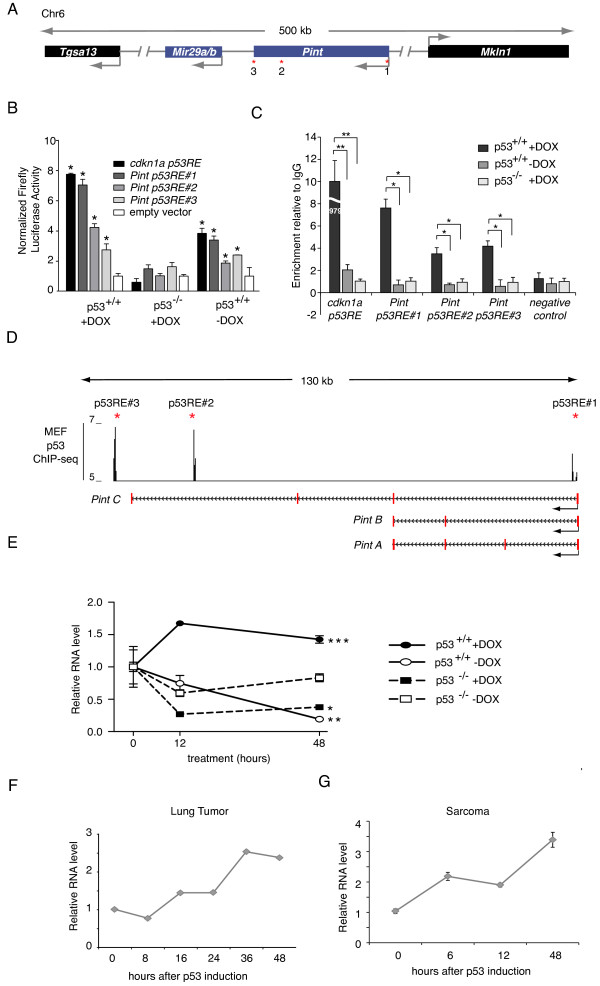
***Pint *is a p53-regulated long intergenic non-coding RNA (lincRNA)**. **(A) **Schematic representation of the *Pint *genomic locus. Asterisks represent p53 response elements (p53REs). **(B) **Relative firefly luciferase expression driven by genomic sequences containing p53REs in p53-restored p53^LSL/LSL ^(p53^+/+^) or p53^LSL/LSL ^(p53^-/-^) cells treated with doxorubicin. Values were normalized by Renilla levels and are the mean ± standard deviation (SD) of three biological replicates. Asterisks represent significant differences determined by *t*-test relative to the same plasmid transfected in doxorubicin (DOX)-treated p53^-/-^. **(C) **Effect on *Pint *p53RE-1, p53RE-2, and p53RE-3, *Cdkn1a *p53RE, or an irrelevant region (control) of p53 chromatin immunoprecipitation (ChIP) enrichment in p53-restored p53^LSL/LSL ^(p53^+/+^) or p53^LSL/LSL ^(p53^-/-^) cells treated with doxorubicin (+DOX) or left untreated (-DOX). Enrichment values are relative to input and the mean ± SD of three biological replicates. Asterisks represent statistical significant differences from the control as determined by *t*-test (*P<0.05, **P<0.01). **(D) **(Top) p53 ChIP sequencing (ChIP-seq) peaks from mouse embryonic fibroblasts (MEFs) treated with doxorubicin [21]. Positions of p53REs are indicated by red asterisks. (Bottom) *Pint *variants identified by 5' and 3' rapid amplification of cDNA ends (RACE) cloning. **(E) ***Pint *levels detected by quantitative real time RT-qPCR in p53-restored p53^LSL/LSL ^(p53^+/+^) or p53^LSL/LSL ^(p53^-/-^) cells treated with 150 nM doxorubicin (+DOX) or left untreated (-DOX) for the indicated time (values represent the mean ± SD of three biological replicates, and asterisks represent significant differences of *Pint *level at 48 hours relative to the doxorubicin-treated p53^-/- ^cells). **(F,G) ***Pint *levels at different times after p53 restoration in **(F) **lung tumor **(G) **and sarcoma cell lines. Values are the mean ± SD of four replicates.

Despite p53 being one of the most studied biological molecules, it has only recently become clear that p53 directly regulates numerous small and large non-coding RNAs [2-4]. In addition, the nature of these transcripts and the role that they play in this tumor suppressor pathway remains relatively unexplored. By using custom tiling microarrays, we previously identified multiple polyadenylated non-coding transcripts that were induced upon expression of p53 in mouse model systems [3]. In that study, we showed that one of the most significantly induced non-coding RNAs, previously named lincRNA-Mkln1 (which from this point we refer to as *Pint *(p53-induced non-coding transcript)), is generated from an intergenic region located on chromosome 6 (Figure 1A; see Additional file 1: Figure S1A). To investigate the regulation of this genomic region by p53, we searched for p53 binding motifs using a method that scores genetic conservation based on the evolutionary substitution pattern inferred for the binding site locus [19]. We found three putative p53 response elements (p53RE-1, p53RE-2, and p53RE-3) inside this region with a high Pi LOD score (>110) (Figure 1A; see Additional file 2).

To experimentally test the biological activity of these regulatory elements, we first cloned the genomic regions of p53RE-1, p53RE-2, and p53RE-3 into a reporter vector, and transfected them into p53-reconstituted (p53^+/+^) or non-reconstituted (p53^-/-^) p53^LSL/LSL ^mouse embryonic fibroblasts (MEFs) to test the reporter-gene induction in the presence or absence of p53. The tested sequences were able to drive transcription of the reporter gene in the presence but not in the absence of p53, with the transcriptional induction being even higher when the p53^+/+ ^cells were treated with the DNA-damaging drug doxorubicin (Figure 1B).

Next, to verify the activity of the p53REs in *Pint *locus, we performed p53 chromatin immunoprecipitation (ChIP), which showed specific and robust binding of p53 to all three predicted p53REs in the endogenous locus upon doxorubicin-induced DNA damage in p53^+/+^, but not p53^-/- ^cells (Figure 1C).

To further confirm our observations, we analyzed previously published p53 ChIP sequencing (ChIP-seq) data from mouse embryonic stem cells (mESCs) (total and phosphorylated p53) [20] and MEFs (total p53) [21]. In mESCs, we identified ChIP-seq peaks of total and phosphorylated p53 after doxorubicin treatment at positions corresponding to *Pint *p53RE-1 and p53RE-2, but not at the position corresponding to p53RE-3 (see Additional file 1, Figure S1), suggesting that p53RE-3 activity may be cell type-dependent. The previously published p53 ChIP-seq data from MEFs showed specific peaks at the *Pint *p53RE-1, p53RE-2, and p53RE-3 locations in doxorubicin p53 wild-type but not p53-null MEFs, in agreement with our results (Figure 1D). Together, these data confirm that the *Pint *genomic locus is controlled by p53, which directly binds to the harbored regulatory sequences.

To better define the length and structure of the transcripts produced in this p53-regulated locus, we carried out 5' and 3' rapid amplification of cDNA ends (RACE) cloning from doxorubicin-treated MEFs. We detected a transcript of 1157 nucleotides and 4 exons, similar to the annotated EST BC145649, isoform that we named *Pint A*. Additionally, we cloned two shorter transcripts of 516 (*Pint B*) and 659 (*Pint C*) nucleotides, which share three and two exons, respectively, with the longer *Pint A *variant (Figure 1D; see Additional file 1: Figure S2A).

To obtain additional information on *Pint *transcript structure, we analyzed publicly available RNA-seq data from mouse heart, thymus, and small intestine by using the Cufflinks method for transcript assembly (see Additional file 1: supplementary methods). This analysis predicted six different RNA isoforms in this region, two of which correspond to *Pint A *and *Pint B *variants (see Additional file 1: Figure S2A). *Pint C *was not detected by the RNA-seq analysis, which could be due to the different cell type used for RACE cloning. Interestingly, one of the experimentally validated p53REs (p53RE-1), is located at the 5' end of all detected transcripts, in agreement with the transcriptional activation of these response elements by p53 (see Additional file 1: Figure S2A). The other two p53 sites (p53RE-2 and p53RE-3) are respectively 100,000 and 120,000 bp further downstream (Figure 1D). Additionally, ChIP-seq data from MEFs showed that p53RE-1 overlaps with a peak for H3K4me3, the chromatin mark associated with active promoters, while p53RE-2 and p53RE-3 are also enriched in H3K4me1 (see Additional file 1: Figure S2A), suggesting that the two distal sites could act as enhancers.

Next, to characterize the non-coding nature of the cloned RNAs, we analyzed the coding potential across all full-length isoforms identified. All the potential ORFs found in the transcripts are small (< 100 amino acids) and do not contain evolutionary conserved codons (Codon Substitution Frequencies scores < -205), strongly suggesting a lack of protein-coding capacity [22].

We focused our studies on variant *Pint A *(1157 nt), which is the longest of the cloned isoforms and the one with the highest expression level (see Additional file 1, Figure S2B). Analysis of *Pint A *expression across a panel of normal mouse tissues showed that it is ubiquitously expressed (see Additional file 1: Figure S2C).

We next tested the expression of *Pint *in different mouse cell types at different time points after induction of p53 by doxorubicin-induced DNA damage, including p53^+/+ ^and p53^-/- ^MEFs (Figure 1E), and K-RAS lung tumor and sarcoma cells (Figure 1F,G; see Additional file 1: Figure S2D) after genetic restoration of the *p53 *gene [23]. In all cell lines tested, levels of *Pint *increased significantly in a temporal manner upon p53 induction.

Next, to further confirm the regulation of *Pint *by p53, we depleted p53 by small interfering RNA (siRNA) treatment in p53-restored p53^LSL/LSL ^MEFs (which are functionally equivalent to p53^+/+ ^MEFs [23]), and transfected a non-targeting siRNA as control. Inhibition of p53 resulted in a decrease of *Pint *levels, whereas the control siRNA had no effect (see Additional file 1: Figure S2B). We therefore conclude that *Pint *expression is induced in a p53-dependent manner.

Collectively, our results show that *Pint *is a ubiquitously expressed lincRNA, which has several isoforms and is transcriptionally regulated by p53.

### *Pint *modulates cell survival and proliferation

To elucidate the biological role of *Pint*, we attempted to perform RNA interference (RNAi)-mediated loss of function studies. To that end, we designed multiple *Pint*-targeting siRNAs and short hairpin RNAs (shRNAs), and transfected or transduced cells to deplete the lincRNA levels. However, these strategies were unsuccessful in lowering the levels of *Pint *(data not shown), which prompted us to use an alternative approach.

**Figure 2:**
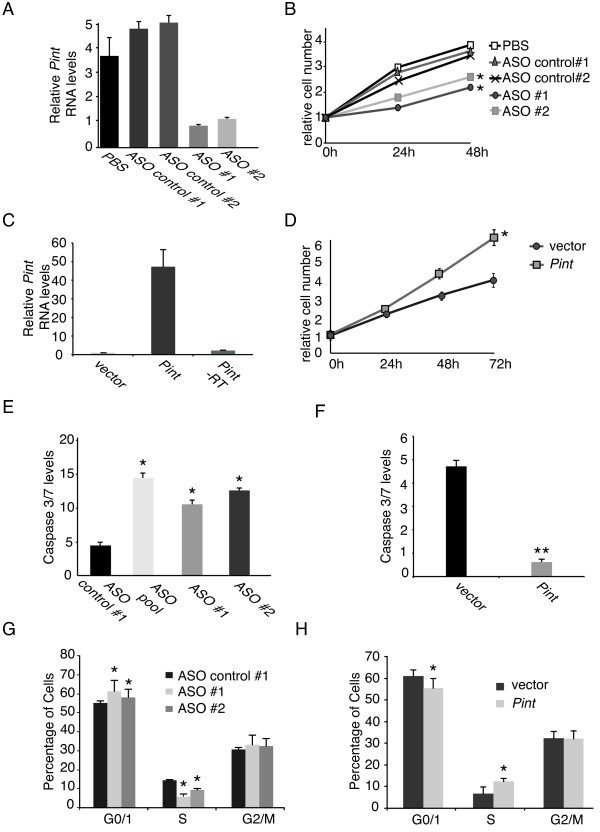
***PINT *modulates cell proliferation and apoptosis**. **(A) **Inhibition of *Pint*. *Pint *levels were detected by quantitative real time (RT-qPCR) in p53-restored doxorubicin-treated p53^LSL/LSL ^MEFs 36 hours after transfection with two *Pint*-specific anti-sense oligonucleotides (ASOs) (ASO1 and ASO2), two control ASOs (control ASO -1 and -2), or a blank (PBS) control, and 12 hours of doxorubicin treatment. Values normalized to *Gapdh *and are the mean ± SD of three replicates. **(B) ***Pint *positively regulates cell proliferation. Relative number of p53-restored p53^LSL/LSL ^mouse embryonic fibroblasts (MEFs) transfected with ASOs for *Pint *inhibition, and treated with doxorubicin from 24 h post-transfection. Cell numbers are determined by MTS assay. Values are mean ± SD of three replicates. **(C) **Overexpression of *Pint*. *Pint *levels where measured like in **(A) **in p53-restored doxorubicin-treated p53^LSL/LSL ^MEFs 36 hours after transfection and 12 hours of doxorubicin treatment with *Pint *A isoform expressing plasmid or an empty plasmid as control. **(D) ***Pint *positively regulates cell proliferation. Cells were transfected as in **(C) **and treated with doxorubicin from 24 hours post-transfection. **(E,F)**. Negative effect of *Pint *on apoptosis induction. Apoptosis levels were determined by quantification of caspase 3/7 levels after **(E) **inhibition or **(F) **overexpression of *Pint *in p53-restored p53^LSL/LSL ^MEFs treated with doxorubicin. Values are the mean ± SD of three replicates. **(G,H)**. Effect of *Pint *on cell cycle regulation. Relative cell numbers in each cell cycle phase were determined by fluorescence-activated cell sorting (FACS) of bromodeoxyuridine (BrdU) incorporation and propidium iodide (PI) staining of p53-restored p53^LSL/LSL ^MEFs treated as in **(A) **or **(C)**. Percentages of cells in each phase are represented and values are the mean ± SD of three replicates.

We then designed anti-sense oligonucleotides (ASOs) with special modifications to target *Pint *for degradation by RNaseH [24]. By independently transfecting two different *Pint*-targeting ASOs we were able to obtain a significant decrease (>75%) in *Pint *levels compared with transfections using two independent control ASOs or in the absence of oligo transfection (PBS) (Figure 2A; see Additional file 1: Figure S3A). Interestingly, we were able to deplete all three *Pint *isoforms (data not shown), supporting the notion that the inhibition by ASOs occurs at the level of pre-spliced RNA [25].

Next, to assess the effect of *Pint *downregulation, we treated p53-restored p53^LSL/LSL ^MEFs with two independent *Pint*-targeting ASOs or with two independent control ASOs, and monitored cell proliferation at 24 and 48 hours after transfection, while treating the cells with doxorubicin to induce the p53 response (Figure 2B). There was a significant decrease in proliferation of cells depleted of *Pint *by the two specific ASOs compared with the cells treated with either of the two ASO controls or compared with the untransfected cells (Figure 2B). Conversely, when *Pint *(isoform A) was transiently overexpressed using a plasmid under the control of a cytomegalovirus promoter (Figure 2C), cell proliferation was increased compared with the cells transfected with the empty plasmid (Figure 2D). Similarly, stable overexpression of *Pint *by retroviral infection had a positive effect on cell proliferation rate (data not shown). Interestingly, we also found a slight effect on proliferation after *Pint *depletion and overexpression in the absence of doxorubicin-induced DNA damage, although this was not as significant as in the presence of doxorubicin (see Additional file 1: Figure S3B,C). We thus concluded that *Pint *is a positive regulator of cell proliferation.

To determine how cell proliferation is modulated by *Pint*, we investigated different aspects of the cellular phenotype. When *Pint *was depleted and cells were treated with doxorubicin to induce DNA damage, there was a significant increase in the number of apoptotic cells (Figure 2E). Consistent with these results, *Pint *overexpression resulted in the opposite phenotype, decreasing cellular apoptosis (Figure 2F). There was also a slight but significant effect on cell cycle regulation. Transfection of the specific *Pint*-targeting ASOs caused a decrease in the fraction of S-phase cells and a concomitant increase in the fraction of cells in G1 (Figure 2G), whereas *Pint *overexpression caused the opposite effect (Figure 2H). These data suggest that, under DNA-damaging conditions, *Pint *affects both induction of apoptosis and regulation of the cell cycle.

Next, to determine whether the aforementioned effects were cell type-specific, we performed similar experiments in the mouse lung tumor cell line LKR [3]. There was a significant decrease in cell proliferation following *Pint *depletion in these cells by doxorubicin-induced DNA damage (see Additional file 1: Figure S3D,E), whereas lincRNA overexpression caused the opposite effect (see Additional file 1: Figure S3F,G). Inhibition of *Pint *in doxorubicin-treated 3T3 cells caused a similar effect on cell proliferation and apoptosis induction (see Additional file 1: Figure S3H to K). Furthermore, depletion of *Pint *in these cells affected their ability to grow independently of attachment and to form colonies independently of cell-cell contact (see Additional file 1: Figure S3L,M), opposite to the effect caused by *Pint *overexpression (see Additional file 1: Figure S3N).

We therefore concluded that *Pint *positively regulates cell viability and proliferation at different levels, including induction of cellular apoptosis and regulation of the cell cycle, both in the presence and absence of DNA damage.

### *Pint *regulates the expression of genes involved in cell proliferation and survival, including genes of the p53 pathway

**Figure 3:**
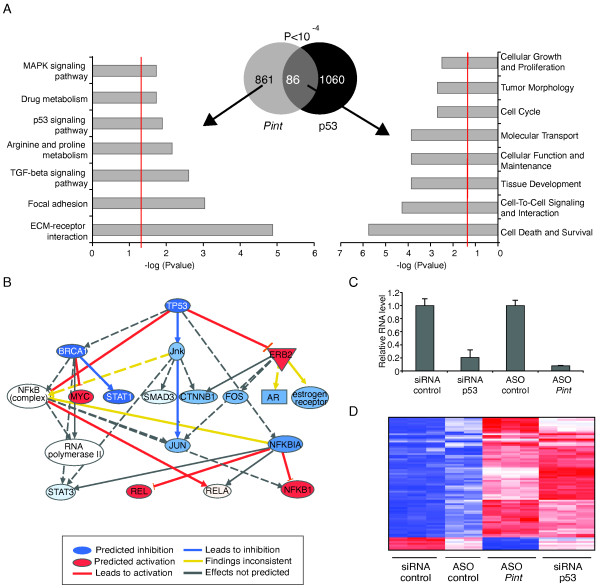
***PINT *regulates the expression of genes involved in cell proliferation and survival, including genes of the p53 pathway**. **(A) **(Left) Significant KEGG (Kyoto Encyclopedia of Genes and Genomes) pathways enriched in the 947 genes regulated by *Pint*. Center: Venn diagram representing the number of genes affected by *Pint *(947), and p53 knockdown in p53-restored doxorubicin-treated p53^LSL/LSL ^mouse embryonic fibroblasts (MEFs) (B > 3). P-value represents the probability associated with the overlap between both gene sets (86 genes). (Right) Significant biologic functions of genes co-regulated by *Pint *and p53. The red line represents *P *= 0.05. **(B) **Predicted p53 regulatory network based on the fold change of genes affected by *Pint *depletion (ingenuity pathway analysis). **(C) **Relative *Pint *RNA levels after *Pint *or p53 depletion. Values are normalized to *Gapdh *and are the mean ± SD of four replicates. **(D) **Genes commonly affected by *Pint *and p53 depletion (B > 3). Colors represent transcripts above (blue) or below (red) the global median, scaled to twofold activation or repression, respectively.

Given the role of *Pint *in cell survival and proliferation, we wanted to investigate the effect of the lincRNA on gene expression. We transfected p53-restored p53^LSL/LSL ^MEFs with a pool of ASOs to deplete *Pint *expression or with a non-targeting ASO as control, then treated the cells with doxorubicin to induce DNA damage, and extracted total RNA for microarray analysis in triplicate. We identified 947 genes affected by lincRNA inhibition (B > 3) (see Additional file 1,: Figure S4A; see Additional file 3). Gene Ontology analysis of these genes identified significant enrichment in pathways relevant for signaling, proliferation, and survival, including extracellular matrix (ECM)-receptor interaction and transforming growth factor (TGF)-β, mitogen-activated protein kinase (MAPK), or p53 signaling pathways (Figure 3A; see Additional file 3). In agreement with this observation, the most significant biological functions of *Pint*-regulated genes included cancer, cellular movement, cellular growth and proliferation, cell death and survival, and organism development (see Additional file 1: Figure S4B).

To independently validate the microarray findings, cells were transfected with two different *Pint*-targeting ASOs or two control ASOs, and the levels of 15 mRNAs were determined by quantitative real time (RT-qPCR). This experimental validation confirmed the microarray results for 14 of 15 genes (93%), including the downregulation of *Tgfβ1, Serpina3n, Nkx2-9*, and *Il1r1*, and upregulation of *Gadd45b *and *Egr2*, among others (see Additional file 1, Figure S4C).

Interestingly, genes affected by *Pint *inhibition did not include any of the six neighboring genes located 250 kb upstream or downstream of the *Pint *locus. Furthermore, upon *Pint *depletion, we did not observe any change in the levels of the microRNA mir29a/b, encoded downstream of *Pint*, which we determined by RT-qPCR on the small RNA fraction of cells (data not shown). Therefore, our data suggest that although *Pint *depletion affects the expression of hundreds of genes, *Pint *does not act on genes that are proximally located to it.

The direct transcriptional regulation of *Pint *by p53 strongly suggests a functional relationship between the two. This relationship was confirmed by the presence of the p53 pathway as one of the cellular pathways most affected by *Pint *inhibition (Figure 3A). Moreover, the microarray data analysis predicted p53 as one of the upstream regulators of genes affected by *Pint *(B > 5, *P *= 4.20 × 10^-13^) (Figure 3B; see Additional file 4).

To further explore the relationship between *Pint *and p53, we treated cells with p53 siRNA or a control siRNA, and subjected the extracted RNA to microarray analyses (Figure 3C). As expected, the analyses showed that hundreds of genes were affected by p53 depletion (1,146 genes, B > 3), including most of the well-known p53 target genes such as *Cdkn1A*, *Fas*, and *Perp *(see Additional file 5).

Next, to detect genes co-regulated by p53 and *Pint*, we compared the genes affected by p53 depletion with those affected by *Pint *depletion. Interestingly, a significant subset of the genes affected by *Pint *inhibition was similarly affected by p53 inhibition (86 genes, B > 3, *P *= 1.5 × 10^-5^) (Figure 3A,D). These genes were enriched in functional terms that include cellular apoptosis and cell cycle regulation (Figure 3A), and secondary targets of p53, such as *Ikbke, Dgka, Adam8*, and *Serpine2 *(see Additional file 6). These results confirmed that *Pint *gene regulation comprises part of the p53 transcriptional response.

In addition to the transcription factor p53, other upstream regulators are predicted for *Pint*-regulated genes, including the cytokine tumor necrosis factor α, the transcription regulator nuclear factor κB1A or the tumor growth factor -β1 (see Additional file 4). We therefore investigated the genes that are regulated by *Pint *but not p53. The most significant biological functions of the genes regulated specifically by *Pint *include cell death, response to hypoxia, and vasculogenesis (see Additional file 1: Figure S4D). By contrast, the top biological functions of the genes regulated by p53 but not *Pint *are segregation of chromosomes, mitosis, and cell cycle progression (see Additional file 1: Figure S4E). These results confirm that *Pint *is involved in biological processes related to survival and invasion, which are different from those of the p53 core response. In addition, the *Pint*-independent component of the p53 pathway is clearly enriched in cell cycle regulatory genes.

Taken together, our data strongly suggest that *Pint *plays an important role in gene regulation via a *trans*-mediated mechanism, modulating cellular pathways that are crucial for cell survival and proliferation, including genes of the p53 pathway.

### *Pint*, a nuclear RNA that interacts with Polycomb repressive complex 2

**Figure 4:**
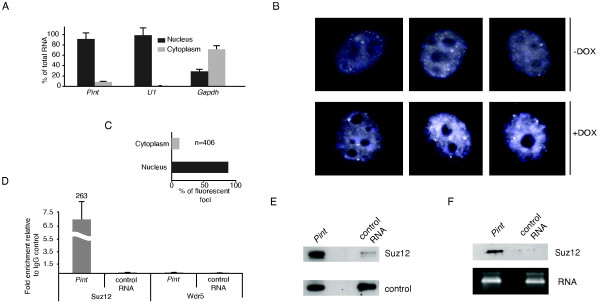
***Pint *is a nuclear long intergenic non-coding RNA (lincRNA) that interacts with PRC2**. **(A) ***Pint *subcellular localization. Percentage of total RNA found in the nuclear and cytoplasmic fractions of p53-restored doxorubicin-treated p53^LSL/LSL ^mouse embryonic fibroblasts (MEFs) determined by quantitative real time(RT-qPCR). **(B) **Single-molecule visualization of *Pint*. RNA fluorescent *in situ *hybridization (FISH) of *Pint *in 3T3 cells untreated (-DOX) or treated (+DOX) with doxorubicin. **(C) **Quantification of the relative subcellular distribution of *Pint *FISH foci. **(D) **Physical association between *Pint *and PRC2 after chemical crosslinking of cells. Suz12 or Wdr5 were immunoprecipitated from nuclear extracts of formaldehyde-crosslinked p53-restored doxorubicin-treated p53^LSL/LSL ^MEFs, and associated RNAs were detected by RT-qPCR. The relative enrichment was calculated as the amount of RNA associated with Suz12 or Wdr5 IP relative to the IgG control. *Gapdh *mRNA was used as control RNA. **(E) ***In vitro *interaction of *Pint *with Polycomb repressive complex 2 (PRC2). Protein associated with biotinylated *Pint *or the anti-sense (control) RNA incubated with nuclear extracts. The bottom band shows the crossreaction of the antibody with a non-specific binding protein. **(F) **Direct binding of PRC2 and *Pint*. Protein bound to *Pint *or anti-sense (control) RNA when incubated with purified PRC2.

We next investigated the mechanism by which *Pint *regulates gene expression. We first analyzed the subcellular localization of *Pint *by RT-qPCR in nuclear versus cytoplasmic fractions, and found that at least 80% of the *Pint *RNA was present in the cell nucleus (Figure 4A). We further confirmed this observation by single-molecule RNA fluorescence *in situ *hybridization (FISH) to detect individual molecules of *Pint *in 3T3 MEFs. The analysis showed that more than 85% of the *PINT *foci were present in the cell nucleus (Figure 4B,C).

Because many lincRNAs have been found to be associated with nuclear protein complexes [7,8,10], we hypothesized that this could be the case for *Pint*. Interestingly, a transcript that we found to correspond to *Pint *was previously identified in a genome-wide screen by RNA immunoprecipitation sequencing (RIP-seq) for PRC2-interacting RNAs in mouse embryonic stem cells [26]. Moreover, analysis of the *Pint *A sequence revealed the presence of 10 Ezh2-interacting motifs [27], a larger number than would be expected by chance (*P *< 0.05). Therefore, we decided to test the interaction between *Pint *and PRC2.

To do so, we performed crosslinking followed by RIP in doxorubicin-treated MEFs using an antibody specific for the Suz12 subunit of PRC2 or, as control, an antibody against WD repeat domain 5 (Wdr5), a protein associated with the mixed lineage leukemia (MLL) chromatin activator complex. We found a highly significant enrichment of *Pint *in PRC2 immunoprecipitates, whereas no *Pint *enrichment was seen with the Wdr5 antibody or control IgG (Figure 4D).

To further confirm the interaction between *Pint *and PRC2, we carried out RNA pulldown experiments using *in vitro *synthesized and biotin-labeled *Pint *RNA, and nuclear extracts of doxorubicin-treated MEFs. *Pint *was able to pull down PRC2, as detected by western blotting using an anti-Suz12 antibody, whereas only negligible levels of Suz12 were bound by the anti-sense RNA sequence used as control (Figure 4E).

In addition, to determine whether the interaction between *Pint *and PRC2 was direct or mediated by other factors, we performed RNA pulldown experiments with *in vitro *biotinylated *Pint *RNA and purified PRC2. We found that *Pint *was able to interact with the recombinant purified PRC2, unlike the control RNA (Figure 4F), suggesting that *Pint *directly binds to PRC2.

In conclusion, our data show that *Pint *is mainly localized to the cell nucleus, and directly interacts with PRC2.

### *Pint *affects gene expression by regulating PRC2 occupancy of specific genes for repression

**Figure 5:**
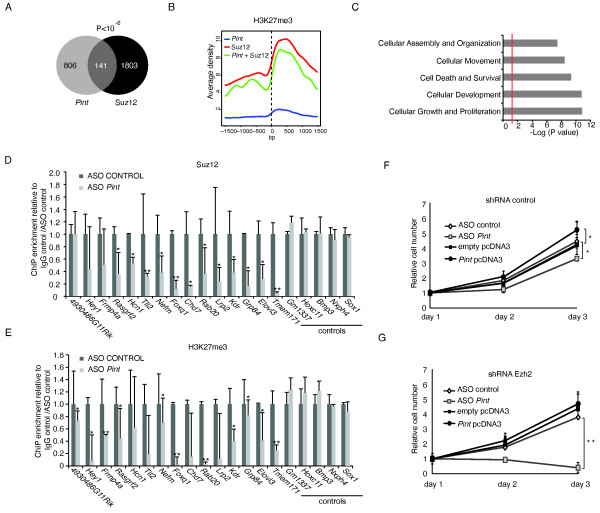
***PINT *is required for Polycomb repressive complex 2 (PRC2) targeting of specific genes for repression**. **(A) **Representation of the number of genes that are regulated by *Pint *in p53-p53^LSL/LSL ^mouse embryonic fibroblasts (MEFs) (B > 3) (left) and/or reported as bound by Suz12 [31]. The *P*-value represents the probability associated with the overlap between both gene sets. **(B) **Mean H3K27me3 ChIP-seq signal around the transcription start site (TSS) of genes regulated by *Pint *but not bound by Suz12 (blue)l genes bound by Suz12 but not regulated by *Pint *(red)l and genes regulated by *Pint *and bound by Suz12 (green) in mouse embryonic stem cells (mESCs) [9]. **(C) **The most significant functions of genes regulated by *Pint *and bound by Suz12. **(D,E) **Relative **(D) **Suz12 or **(E) **H3K27me3 enrichment in promoter regions of H3K27me3-regulated genes [32] in doxorubicin (DOX)-treated p53-reconstituted p53^LSL/LSL ^MEFs treated with *Pint *antisense oligonucleotides (ASOs) or control ASOs determined by chromatin immunoprecipitation-quantitative PCR (ChIP-qPCR). Enrichment values are relative to IgG and the ASO control, and are the mean ± **SD **of three replicates. For each gene, asterisks indicate the significant difference between ASO *Pint *and ASO control: *P < 0.05; **P < 0.001**(F) **Relative cell numbers of control short hairpin RNA (shRNA) stable 3T3 MEFs transfected with the indicated ASOs or plasmids. **(G) **Relative cell numbers of Ezh2 shRNA stable 3T3 MEFs treated as in **(F)**. Values are the mean ± SD of three replicates. *P < 0.05; **P < 0.001 relative to the control transfection.

Several studies suggest that the association of lincRNAs with chromatin complexes such as PRC2 provides regulatory specificity to the complexes by localizing them to genomic DNA targets [3,10,11,28-30]. We therefore hypothesized that *Pint *may act by regulating the binding of PRC2 to certain genomic loci for their repression. Consistent with this hypothesis, we found that a significant number of the genes regulated by *Pint *(141 genes, *P *= 1.4 × 10^-7^) had been previously reported [31] as bound by PRC2 in mESCs (Figure 5A; see Additional file 7). In agreement with this observation, we found this subset of *Pint*-regulated genes to be enriched in H3K27 tri-methylation around their transcriptional start site, whereas the remaining *Pint *-regulated genes showed low H3K27me3 but high H3K4me3 levels (Figure 5B; see Additional file 1: Figure S5A). Interestingly, pathway analysis of these genes identified enrichment in MAPK signaling, ECM-receptor interaction, and TGF-β signaling, consistent with these pathways being affected by *Pint *inhibition (see Additional file 1: Figures S5B and S3A). Furthermore, the most significant biological function of this gene subset is cellular growth and proliferation (Figure 5C).

Next, to further test our hypothesis of *Pint *requirement for PRC2 targeting, we proceeded as follows. We randomly selected a group of 15 genes that (i) we found to be de-repressed by inhibition of *Pint *in p53-restored p53^LSL/LSL ^MEFs (see Additional file 3; see Additional file 1: Figure S5C), and (ii) have been reported as regulated by H3K27me3 in MEFs [32]. We reasoned that these genes might be co-regulated by PRC2 and *Pint*. We then determined the association of PRC2 with these genes by Suz12 ChIP-qPCR in p53-restored p53^LSL/LSL ^MEFs, with or without inhibition of *Pint *(Figure 5D). The ChIP results confirmed that Suz12 occupied all the analyzed genes in the control conditions (see Additional file 1: Figure S5D). Interestingly, the binding of Suz12 to 12 of these loci (80%) was significantly decreased upon *Pint *depletion (Figure 5D; see Additional file 1: Figure S5D), correlating with an increase in their expression (see Additional file 1: Figure S5C). However, there was no change in Suz12 occupancy of genes that are bound by Suz12 but whose expression was unaffected by *Pint *knockdown (Figure 5D, controls; see Additional file 1: Figures S5C, D).

Similarly, we performed ChIP to determine the level of H3K27me3 at these gene promoters under the same experimental conditions. Correlating with the loss of PRC2 binding, there was a significant decrease in H3K27me3 levels in 11 of the 15 (73%) analyzed regions when *Pint *was inhibited (Figure 5E; see Additional file 1: Figure S5E), whereas there were no significant changes at the control genes. Interestingly, in some cases, the changes in H3K27me3 were not as pronounced as the loss of Suz12 binding, probably because of the need for cell division and/or histone demethylase activity to erase the histone mark.

We therefore conclude that *Pint *is required for PRC2 targeting to these genes, which in turn affects their H3K27 methylation levels and expression.

We speculated that if regulation by *Pint *is mediated by its interaction with PRC2, the effect of *Pint *in cell proliferation should be strongly dependent upon PRC2 presence. To test this hypothesis, we generated 3T3 MEFs with stable knockdown of the Ezh2 subunit of PRC2, using shRNA lentiviral transduction, and, as control, we transduced 3T3 MEFs with a non-targeting shRNA. The Ezh2 shRNA stable cell line showed a decrease of around 60% in Ezh2 protein levels compared with the shRNA control cell line (see Additional file 1: Figure S5F).

Next, we transfected both cell lines with a pool of ASOs to deplete *Pint *levels, or with a control ASO (see Additional file 1: Figure S5G,H), and determined their proliferation rate. The proliferation of control cells was negatively affected by *Pint *inhibition (Figure 5F), and interestingly, arrest of proliferation was strongly enhanced when both *Pint *and Ezh2 were depleted in the cells (Figure 5G). In parallel, both cell lines were transfected with a plasmid overexpressing *Pint *or a control plasmid, and their proliferation rate was determined. Although *Pint *overexpression induced proliferation of the control cells, it had no effect on the Ezh2-depleted cells (Figure 5F,G). These results suggest that the biological function of *Pint *requires PRC2, indicating a functional relationship between *Pint *and Ezh2.

Taken together, these data indicate that *Pint *is required for the targeting of PRC2 to some genes for repression, which in turn affects the proliferative state of the cells.

### Human *PINT *is a putative tumor suppressor lincRNA

The role that *Pint *plays in gene regulation and in the p53 tumor suppressor pathway motivated us to explore whether a *Pint *human ortholog exists. We found that the *PINT *human syntenic genomic region in chromosome 7 also encodes a non-coding RNA annotated as FLJ43663. Comparison of human and mouse sequences identified that the highest homology between the two lincRNAs resides at the 5' end of their sequences (see Additional file 1: Figure S6A,B). We therefore hypothesized that, similarly to mouse *Pint*, human *PINT *is regulated by p53.

**Figure 6:**
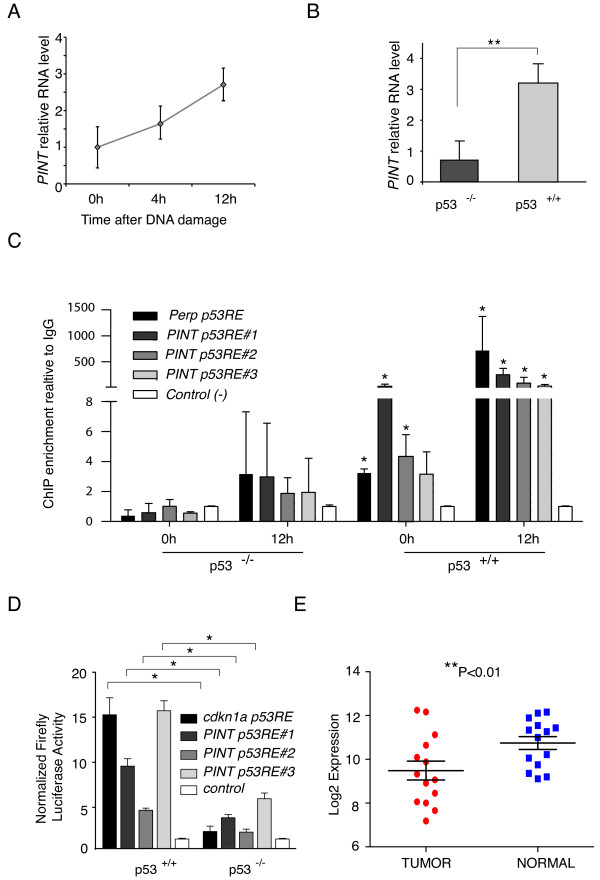
**Human *PINT *is a p53-regulated long intergenic non-coding RNA (lincRNA) downregulated in colorectal cancer**. **(A) ***PINT *is induced by 5-fluoracil (5-FU)-induced DNA damage. Relative *PINT *expression levels in HCT116 cells treated with 5-FU for the indicated times. **(B) ***PINT *is regulated by p53. Relative *PINT *levels in p53^+/+ ^or p53^-/- ^matched HCT116 cells treated with 5-FU for 12 hours. Values are the mean ± SD of three replicates normalized to *GAPDH*. Asterisks represent significant difference between conditions. **(C) **p53 binds to p53 response elements (p53REs) in the *PINT *locus upon 5FU-induced DNA damage. Relative chromatin immunoprecipitation (ChIP) enrichment of p53 to the indicated regions in p53^+/+ ^or p53^-/- ^matched HCT116 cells after the indicated times of treatment with 5-FU. Binding to the *PERP *promoter was included as a positive control and binding to an irrelevant genomic region as the negative control. Enrichment values are relative to IgG and to the negative control for each treatment condition. The mean ± SD of three biologic replicates of a representative experiment is shown, and the significant differences relative to the control are indicated with asterisks. **(D) **p53 drives the transcription of *PINT*. Relative firefly luciferase expression driven by the indicated genomic sequences with p53REs in p53^+/+ ^or p53^-/- ^matched HCT116 cells after treatment with 5-FU. Values were normalized to Renilla levels and are the mean ± SD of three replicates.**(E) ***PINT *is downregulated in colorectal tumors. *PINT *relative expression levels in colorectal cancer samples and normal peripheral tissue.

To test this, we first analyzed the expression of *PINT *by qRT-PCR in p53^+/+ ^and p53^-/- ^matched HCT116 human colorectal cancer cell lines [33], and found that *PINT *was induced in p53^+/+^, but not p53^-/- ^cells when treated with the DNA-damaging drug 5-fluorouracil (5-FU) (Figure 6A,B). Next, to determine the functionality of the three conserved p53REs identified in the mouse and human *PINT *(see Additional file 2), we performed p53 ChIP on the human cells. The p53 protein was found to bind to the three human p53REs upon doxorubicin-induced DNA damage, but not in the absence of treatment (Figure 6C). As control, we also included p53^-/- ^cells, in which we did not detect any p53 ChIP enrichment (Figure 6C).

To further confirm the regulation of *PINT *by p53, we cloned the human genomic sequences harboring each of the three p53REs into a plasmid containing a reporter gene. The three sequences were able to induce expression of the reporter gene when transfected into p53 wild-type, but not p53-null cells (Figure 6D).

Together, our data indicate that *PINT *is a *bona fide *p53 transcriptional target, with conserved regulation across mammalian species.

Given the crucial role that p53 plays in cancer, we speculated that *PINT *expression might be altered in primary tumors. To test this hypothesis, we analyzed *PINT *levels in tumor tissue and matched normal tissue samples from 14 surgical patients with colorectal cancer (stages I to III) (see Additional file 8). Intriguingly, we found a significant (*P *< 0.01) downregulation of *PINT *in colorectal tumors compared with normal tissue (Figure 6E), suggesting a potential role of the lincRNA as a tumor suppressor.

**Figure 7:**
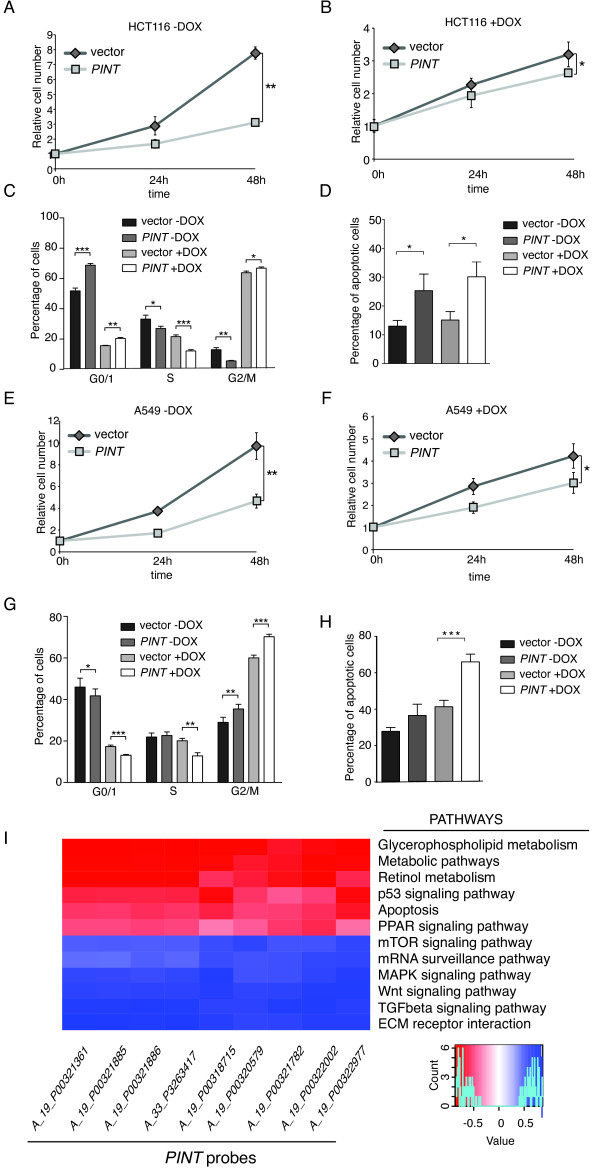
**Human *PINT *inhibits tumor cell growth**. **(A.B)**. Relative number of *PINT*-overexpressing HCT116 cells (*PINT*) or control cells (vector) that were **(A) **left untreated or **(B) **treated with 500 nM doxorubicin (DOX), as determined by MTS assay. **(C) **Relative number of cells of HCT116 stable cell lines in each phase of the cell cycle. Cells were treated as for **(A) **and **(B) **for 12 hours, and cell cycle phases were determined by fluorescence-activated cell sorting (FACS) of bromodeoxyuridine (BrdU) incorporation and propidium iodide (PI) staining. **(D) **Percentage of apoptotic cells in HCT116 stable cell lines treated as in **(C)**, determined by quantification of annexin V-positive cells. **(E,F)**. Relative number of *PINT*-overexpressing A549 cells (*PINT*) or control cells (vector) that were **(E) **left untreated or were treated with 500 nM doxorubicin **(F), as **determined by MTS assay. **(G) **Relative number of A549 stable cell lines in each phase of the cell cycle determined as in **(C)**. **(H) **Relative number of cells of A549 stable cell lines undergoing apoptosis, determined as in **(D)**. **(I) **Correlation of *PINT *with KEGG (Kyoto Encyclopedia of Genes and Genomes) pathways. Significant correlation coefficients between the indicated cellular pathways and *PINT *microarray probes.

To explore this hypothesis, we investigated in more detail the role of *PINT *in human cells. We first stably overexpressed *PINT *in the HCT116 colon cancer cell line by retroviral infection (see Additional file 1: Figure S6C) and assessed the proliferation rate. Interestingly, cells overexpressing *PINT *showed a significant decrease in their growth rate compared with the control cells in either the presence or absence of doxorubicin-induced DNA damage (Figure 7A,B). This decrease in proliferation was confirmed by the cell cycle profile analysis. Compared with control cells, *PINT *-overexpressing cells had a lower percentage of cells in S phase in the absence of doxorubicin-induced DNA damage (Figure 7C), whereas upon doxorubicin treatment, *PINT*-overexpressing cells showed more pronounced cell cycle arrest, appearing as a significantly lower number of cells in S phase and higher number in G1/0 and G2/M phases (Figure 7C). Subsequently, we quantified the apoptosis levels in these stable cell lines, and found increased apoptosis when *PINT *was overexpressed both in the presence and absence of doxorubicin-induced DNA damage (Figure 7D). We concluded that *PINT *has a negative effect on proliferation and survival of HCT116 cells.

Next, to test whether the observed effects of *PINT *overexpression are specific to HCT116 cells, we generated a stably *PINT*-overexpressing A549 lung adenocarcinoma cell line (see Additional file 1: Figure S6D), and subjected the cells to similar analyses, determining their proliferation rate, cell cycle profile, and apoptosis levels. Overexpression of *PINT *in A549 cells caused decreased proliferation (Figure 7E,F) with an increased number of cells arrested in G2/M phases, both in the presence and absence of doxorubicin-induced DNA damage, but being more pronounced with doxorubicin treatment (Figure 7G) Similarly, enforced *PINT *expression in A549 cells resulted in increased apoptosis, both in the presence and absence of doxorubicin treatment (Figure 7H). Together, these results indicate that *PINT *is a negative regulator of cell proliferation and apoptosis of tumor cells, which is consistent with a role as a tumor suppressor lincRNA.

Next, to obtain further insight into the role of *PINT*, we determined the biological pathways that are associated with *PINT *expression in normal tissues, where it has higher expression. To that end, we obtained gene-expression data from microarrays performed on 23 samples from normal colon (*n *= 14) and rectum (*n *= 8), and a normal colon cell line. The microarrays contained 60,000 probes designed to detect the expression of 27,958 protein-coding genes and 7,419 non-coding RNAs. We selected expression data from nine probes corresponding to *PINT*, and computed the correlation existing between these and the expression of mRNAs grouped in KEGG (Kyoto Encyclopedia of Genes and Genomes) pathways [34]. The analysis showed that *PINT *expression had a significant positive (*r *> 0.5, *n *= 23, *p *< 0.05) or negative (*r *< -0.5, *n *= 23 *p *< 0.05) correlation with a number of pathways similar to those regulated by its mouse ortholog, including the MAPK, Wnt, and TGF-β pathways (positive correlations). and p53, apoptosis, and peroxisome proliferator-activated receptor signaling (negative correlations) (Figure 7I). Thus, our data suggest that *PINT *expression and regulation are conserved between mouse and human. However, their function results in different biological outcomes, possibly reflecting species-specific aspects of cellular pathways.

Collectively, our results show that *PINT *is a lincRNA specifically regulated by p53 in mouse and human cells. In mouse cells, *PINT *promotes proliferation and survival, and functions by regulating targeting of PRC2 to specific genes for repression. The human ortholog, *PINT*, is also transcriptionally regulated by p53, and its expression correlates with similar cellular pathways to those of the mouse lincRNA. However, in contrast to the mouse *Pint*, human *PINT *is a negative regulator of proliferation and survival, and is downregulated in colon cancer, representing a novel tumor suppressor candidate lincRNA.

## Discussion

Although thousands of lincRNAs have been identified in mammalian cells, understanding of lincRNA biology and role in disease remains relatively poor. A common feature of lincRNAs is their fine transcriptional regulation [35,36], which may be key to their specific regulatory roles. The transcription factor p53 has been subjected to thorough scrutiny over the years because of its relevance in cellular homeostasis, but only recently have researchers realized that lincRNAs are an active part of the p53 transcriptional network. Among them is lincRNA-p21, which functions as a transcriptional gene repressor in mouse cells [3], and PANDA, which regulates the expression of pro-apoptotic genes in human fibroblasts [4]. Additionally, the lncRNAs linc-RoR [37] and loc285194 [38] have been reported to be post-transcriptional regulators in this pathway. In this study, we have expanded this knowledge by identifying and characterizing *Pint*, a *bona fide *p53 transcriptional target that acts as negative modulator of the p53 response.

We identified three isoforms of *Pint*, transcribed from an intergenic region in the mouse chromosome 6. These transcripts are likely to be alternatively spliced variants, as they all share the 5' sequences and are regulated by p53. Indeed, *Pint *transcription is closely controlled by p53, which specifically binds to three functional p53REs contained inside the *Pint *genomic locus. While one of the three p53REs is located at the promoter, the other two are several kilobases downstream, and could function as transcriptional enhancers, contributing to the fine regulation of *P*int levels upon p53 activation.

*Pint *levels are finely controlled by p53, but unlike many other known lincRNAs [36], *Pint *is ubiquitously expressed. Even in the absence of p53 activation, *Pint *is relatively robust (see Additional file 1: Figure S2C). This suggests that *Pint *plays a role independently of p53 activation by DNA damage. In fact, inhibition of *Pint *in the absence of DNA damage causes an arrest in cell proliferation, as opposed to the effect of *Pint *overexpression. Furthermore, even in the presence of DNA damage, most of the genes found to be regulated by *Pint *are involved in cellular pathways not directly related to p53. This suggests that *Pint *is necessary for regulation of normal cell growth and proliferation, and in the presence of DNA damage, *Pint *acts as a negative regulator of cell cycle arrest and as a pro-survival molecule, modulating the effect of p53 activation through a negative autoregulatory mechanism.

*Pint *binds directly to PRC2, and is required for the targeting of PRC2 to specific genes for H3K27 tri-methylation and repression. The association of PRC2 with the promoter of these genes is lost when *Pint *is depleted from the cells, resulting in their transcriptional induction. Moreover, the biological effect of *Pint *depletion is strongly enhanced by PRC2 downregulation, whereas *Pint *overexpression has no effect in a PRC2 knockdown background. These results suggest that *Pint *cooperates with PRC2 in the repression of genes required for survival and proliferation.

We found that of the total number of genes affected by *Pint *inhibition, 39% were upregulated and 61% downregulated upon *Pint *knockdown, suggesting that many *Pint*-regulated genes are indirect targets of *Pint*-induced gene repression Interestingly, the top functional terms of the genes downregulated by *Pint *are related to transcription regulation (transcription regulator activity, transcription factor activity, and DNA binding). By contrast, *Pint*-upregulated genes are mostly involved in functions related to extranuclear components of signaling cascades such as pattern binding and polysaccharide binding (see Additional file 1: Figure S4F,G). These data are consistent with a model in which *Pint *modulates the targeting of PRC2 to specific transcription regulators, affecting the gene-expression cascade at its top, and resulting in broad downstream effects.

The precise mechanism by which *Pint *contributes to PRC2 targeting to specific loci remains to be defined. One possibility is that *Pint *binds to genomic sequences, either by Crick-Watson base pairing or DNA-RNA-DNA triple helical structures. The latter has been shown for other non-coding RNAs, resulting in transcriptional repression in the case of the dihydrofolate reductase gene, *DHFR *[39], or in epigenetic silencing in the case of ribosomal genes [40]. Additionally, we cannot exclude the possibility that *Pint *interacts with protein complexes other than PRC2,, acting as an RNA scaffold that brings together additional factors that may determine target specificity. In fact, this has been shown for the lincRNA *HOTAIR*, which interacts with the PRC2 and Lysine-specific demethylase 1 complexes [41].

We have identified the human *PINT *ortholog, which, despite relatively low overall sequence homology, shows several analogies with mouse lincRNA. Human *PINT *is not only transcriptionally induced by p53, but it conserves all three fully functional p53REs present in mouse. *PINT *significantly correlates positively or negatively with the same KEGG pathways that are affected by *Pint *knockdown in mouse cells. Furthermore, similarly to the mouse *Pint*, human *PINT *presents nuclear localization, and has previously been reported to interact with PRC2 [10]. The similarities between murine and human lincRNAs suggest that their study could help infer the molecular principles underlying lincRNA functions with low sequence dependency. Intriguingly, *PINT *appears to be significantly downregulated in primary colon tumors, and its overexpression in human tumor cells inhibits their proliferation. These observations contradict what might be expected based on a simplistic interpretation of the mouse *in vitro *phenotype, and could reflect species-specific aspects of cellular pathways and/or the known intrinsic biological differences between mouse *in vitro *models and human tumor cells [42].

## Conclusions

In summary, we have identified a lincRNA, *Pint*, which establishes a new connection between the tumor suppressor p53 and epigenetic regulation by PRC2. Furthermore, the human ortholog of *Pint *may represent a crucial component of the p53 barrier against cancer.

## Materials and methods

### Cell lines, p53 restoration, and DNA damage induction

Lung tumor-derived cell lines were derived from individual tumors in *KrasLA2/+;Trp53LSL/LSL Rosa26CreERT2 *animals [43]. Sarcomas were isolated when they formed in *Trp53LSL/LSL Rosa26CreERT2 *animals as described previously [23]. p53^LSL/LSL ^MEFs were isolated from embryos of the same mouse strain. For p53 restoration, cultured tumor cell lines were incubated with 500 nM 4-hydroxytamoxifen (Sigma) for the indicated time points, and p53^LSL/LSL ^MEFs were infected with AdenoCre or AdenoGFP virus for 24 hours (at the University of Iowa) at a multiplicity of infection (MOI) of 5. NIH/3T3 MEF cells were purchased from ATCC. The LKR Lung tumor-derived cell line was isolated from individual tumors from *KrasLA2/+ *mice. HTC116 p53^+/+ ^and p53^-/- ^were kindly provided by Dr Vogelstein's laboratory. For DNA damage, cells were treated with 100 to 500 nM doxorubicin hydrochloride (D1515; Sigma) or 385 μM of 5-FU.

### Promoter reporter assays and chromatin immunoprecipitation

*PINT *genomic sequences (about 2,000 bp) flanking p53REs were amplified from human and mouse genomic DNA, and subcloned into pGL3-basic vector (Promega). The TK-Renilla plasmid was used as normalizing control. Firefly and Renilla luciferase activities were measured using the dual luciferase reporter assay kit (Promega) and a FLUOstar Optima luminometer (BMG Labtech). ChIP experiments were performed as previously described [44].

### Rapid amplification of cDNA ends (RACE)

Using TRIzol reagent, total RNA was isolated from NIH/3T3 MEFs treated with doxorubicin hydrochloride for 12 hours. cDNAs were then amplified, and *PINT *isoforms were identified using the First Choice RLM-RACE Kit (Ambion), and followed by DNA sequencing.

### Stable cell line generation

For stable *PINT *overexpression, *PINT *was cloned into the pBABE vector for retrovirus production, then NIH/3T3 MEFs were infected and selected with 1.5 μg/ml of puromycin for 72 h. For generation of Ezh2 shRNA and stable control NIH/3T3 cells, shRNA lentiviral infection was used as previously described [45].

### qPCR primers and Antibodies

The qRT-PCR and ChIP-qPCR primer sequences and antibodies used in this study are listed (see Additional file 1: Supplemental methods).

### siRNAs, anti-sense oligo transfection, and *PINT *transient overexpression

All siRNAs and ASOs used in this study are listed (see Additional file 1: Supplemental methods). All ASOs were designed and provided by ISIS Pharmaceuticals. All were 20 nucleotides in length and were chemically modified with phosphorothioate in the backbone, five 2'-*O*-methoxyethyl residues at each terminus, and a central deoxynucleotide region of 10 residues (5-10-5 gapmer). ASOs were synthesized using an Applied Biosystems 380B automated DNA synthesizer (PerkinElmer Life and Analytical Sciences-Applied Biosystems), and purified as previously described [24]. ASOs were selected from a larger panel of oligos, based on the achieved *PINT *RNA inhibition and an absence of toxicity. ASOs were used independently (ASO-1 and ASO-2) or as a pool (ASO-1 to ASO-4) (knockdown levels for each ASO independently are shown; see Additional file 1: Figure S3A). In all cases, ASOs and siRNAs were transfected at a total concentration of 100 nM with Lipofectamine 2000 (Invitrogen) following the manufacturer's instructions. For transient overexpression experiments, *PINT *sequence was cloned into pcDNA3.

### Microarray analysis

For gene-expression profiling, total RNA was extracted and hybridized to an Affymetrix Mouse Genome 430 2.0 microarray. For human tissue samples, total RNA was hybridized to Agilent SurePrint G3 8x60K microarrays. Data normalization and analysis were performed with GiTools [34] (for more information see (see Additional file 1: Supplemental methods)

### Cell proliferation assays

For proliferation analysis, 2,000 cells were plated per well in 96-well plates and assessed with a CellTiter96 Aqueous Non-Radioactive Cell Proliferation Assay (MTS) Kit (Promega).

For clonogenicity assays, cells were transfected, plated at 10^3 ^to 2 × 10^3 ^cells per well of a six-well plate and grown in normal medium for 10 days. Cells were then fixed and stained with crystal violet. For soft agar colony formation assays, 1 × 10^4 ^and 5 × 10^4 ^cells/ml were plated in a volume of 1 ml 0.3% agar (Ref. 214220, C-35; DIFCO) over 1 ml 0.5% agar base layers in each six-well plate. Cultures were monitored for growth by viewing under an inverted microscope. At the time of maximum colony formation (7 to 21 days of culture), colonies were stained with MTT (Sigma), and digital photgraphs were taken.

### Apoptosis and cell cycle analyses

At 24 hours after transfection, 1 × 10^5 ^cells were plated in 96-well white microplates, and treated for 24 hours with 500 nM doxorubicin. Apoptosis was determined by quantification with caspase-Glo 3/7 reagent (Promega) using a FLUOstar Optima luminometer, and with annexin V fluorescence-activated cell sorting (FACS) with an Apoptosis Detection Kit I (cat-559763; BD Biosciences). For cell cycle analysis, cells were labeled for 3 hours with bromodeoxyuridine (BrdU), and stained with propidium iodide (PI) using a BrdU flow kit(BD Bioscience) and sorted and quantified with a BD FACSCalibur flow cytometer (BD Biosciences). Data represent the mean ± SD of a minimum of three biological replicates.

### Human samples

Samples from patients with colorectal cancer (tumor and normal tissue; see Additional file 8) were obtained by surgical resection at the Municipal Hospital of Badalona, Spain. Tumors were staged in accordance with the American Joint Cancer Committee (AJCC) criteria. The adjacent normal tissue was obtained from areas 20 cm distant from the tumor, and diagnosis of normal mucosa was confirmed histologically. The work was carried out in compliance with the Declaration of Helsinki, and all patients provided signed informed consent.

### Nuclear fractionation and fluorescence *in situ *hybridization

Nuclear fractionation was performed as previously described [3]. RNA FISH for *PINT *detection was performed using a pool of 48 fluorescent probes purchased from Stellaris Biosearch Technologies, following the manufacturer's protocol.

### X-linked RNA immunoprecipitation and RNA pulldown

RNA immunoprecipitation was performed after formaldehyde crosslinking of cells, as described previously [3]. RNA pulldowns were performed as described previously [28]. PRC2 was purchased from BPS Bioscience (catalogue number 3m:51003)

### Statistical analysis

Experimental data are represented as the mean ± SD of a minimum of three biologic replicates and were compared using Student's *t*-test. Significant *P*-values are indicated with asterisks as follows: *P < 0.05, **P < 0.01, and ***P < 0.001.

### Accession numbers

Full-length sequences of *PINT *A, B and C have been deposited in GenBank (accession numbers KC860257, KC860259, KC860258 respectively). All primary data are available at the Gene Expression Omnibus (GSE46272).

## Abbreviations

5-FU: fluorouracil; ASO: Anti-sense oligonucleotide; BrdU: bromodeoxyuridine; ChIP: chromatin immunoprecipitation; ChIP-seq: ChIP sequencing; Ezh2: Enhancer of zeste homolog 2; FACS: fluorescence-activated cell sorting; FISH: Fluorescence *in situ *hybridization; KEGG: Kyoto Encyclopedia of Genes and Genomes; lincRNA: Long intergenic non-coding RNA; Mapk: mitogen-activated protein kinase; MEF: mouse embryonic fibroblast; mESC: mouse embryonic stem cell; ORF: Open reading frame; *PINT*: p53 induced non-coding transcript; PRC2: Polycomb repressive complex 2; RT-qPCR: Quantitative real time PCR; RACE: rapid amplification of cDNA ends; RIP-seq: RNA immunoprecipitation sequencing; RNAi: RNA interference; shRNA: short hairpin RNA; siRNA: small interfering RNA; Suz12: Suppressor of zeste 12; Tgf: transforming growth factor; Wdr5: WD repeat-containing protein 5.

## Competing interests

The authors declare that they have no competing interests.

## Authors' contributions

MH and OMB designed the research; OMB, FM, YS, and JG performed the research; AA, VS, and J R performed data analysis; IM, AN, MM, and JGF contributed to the collection and analysis of data from human samples; SG contributed to design, testing and production of reagents; and MH and OMB wrote the paper.

## Supplementary Material

Additional file 1 **Supplemental materials and methods, and supplementary figures with figure legends and *PINT *sequences**. Click here for file

Additional file 2 **Table S1. p53 response elements (p53REs)**. found in mouse and human *PINT *genomic loci. Click here for file

Additional file 3 **Table S2 Genes affected by *Pint *inhibition**. Click here for file

Additional file 4 **Table S3 Predicted upstream regulators of genes affected by *Pint *knockdown**. Click here for file

Additional file 5 **Table S4 Genes affected by p53 inhibition**. Click here for file

Additional file 6 **Table S5 Genes commonly affected by *Pint *and p53 inhibition**. Click here for file

Additional file 7 **Table S6 Genes regulated by *Pint *and bound by Suz12 **[31]. Click here for file

Additional file 8 **Table S7 Information on the human samples used in this study**. Click here for file
