# Supplementary material for: Pint lincRNA connects the p53 pathway with epigenetic silencing by the Polycomb repressive complex 2
Source: Genome Biol. 2013 Sep 26;14(9):R104. doi: 10.1186/gb-2013-14-9-r104 (PMC4053822; doi:10.1186/gb-2013-14-9-r104)
Supplement: Additional file 1 — Supplemental materials and methods, and supplementary figures with figure legends and PINT sequences. [file gb-2013-14-9-r104-S1.PDF]

SUPPLEMENTAL EXPERIMENTAL PROCEDURES:

Analysis of RNA-seq data

Two replicates of long RNA-seq experiments from ENCODE/Cold Spring Harbor Lab corresponding to mouse heart, thymus and small intestine were downloaded from UCSC genome browser and used as input for *Cufflinks* for transcript assembly and *Cuffmerge* to obtain the presented RNA-seq data track.

Affymetrix microarray hybridization and data analysis

Cells were harvested with TRIzol Reagent (Invitrogen) and RNA extracted according to the manufacturer's instructions followed by purification with the RNeasy Mini-kit (Qiagen, Hilden, Germany). Before cDNA synthesis, RNA integrity from each sample was confirmed on Agilent RNA Nano LabChips (Agilent Technologies). The sense cDNA was prepared from 300 ng of total RNA using the Ambion® WT Expression Kit. The sense strand cDNA was then fragmented and biotinylated with the Affymetrix GeneChip® WT Terminal Labeling Kit (PN 900671). Labeled sense cDNA was hybridized to the Affymetrix Mouse Gene 1.0 ST microarray according to the manufacturer protocols and using GeneChip® Hybridization, Wash and Stain Kit. Genechips were scanned with the Affymetrix GeneChip® Scanner 3000.

Both background correction and normalization were done using RMA (Robust Multichip Average) algorithm [1]. Then, a filtering process was performed to eliminate low expression probe sets. Applying the criterion of an expression value greater than 64 in at least two samples for each experimental condition (siRNA_Control, siRNA_P53, ASO_Control, ASO_*Pint*), 21553 probe sets were selected for statistical analysis. R/Bioconductor [2] was used for preprocessing and statistical analysis. LIMMA (Linear Models for Microarray Data) [3] was used to find out the probe sets that showed significant differential expression between experimental conditions. Genes were

selected as significant using criteria based on a combination of B statistic and log fold-change cut offs specific for each contrasts.

ChiP-seq analysis

For generation of average aggregate plots, the proceseed ChIP-Seq datasets for H3K4me3 y H3K27me3 histone modifications of murine ES cell lines were downloaded from the public database GEO (accession number GSE8024). Briefly, reads were aligned to the reference genome, and the fragment count at any given position was estimated as the number of uniquely aligned reads oriented towards it and within 300 bp (Mikkelsen et al. 2007). Composite profile of the promoters of *PINT* genes, Suz12 genes and *PINT*+Suz12 genes were calculated considering the TSS annotated in the reference genome. The plots obtained show mean ChIP-Seq fragment densities of H3K4me3 and H3K27me3 over all analyzed promoters.

Agilent microarray data analysis

Total RNA from human tissue samples was extracted with TRIzol Reagent (Invitrogen) according to the manufacturer's instructions, followed by purification with the RNeasy Mini-kit (Qiagen, Hilden, Germany). Before cDNA synthesis, RNA integrity from each sample was confirmed on Agilent RNA Nano LabChips (Agilent Technologies). RNA was hybridized to Agilent® SurePrint G3 Human GE 8x60 Microarray catalog # G4858A-028004.

Data normalization and analysis was performed using GiTools application [4]. For the correlation analysis first a z-score median enrichment analysis was carried out using KEGG pathway modules and the microarray data. The obtained z-scores matrix, representing the enrichment level of pathways in the samples, was then attached to an expression level matrix containing the lincRNA probes. With this resulting matrix a Pearson’s correlation coefficient analysis was performed.

Functional and pathway analysis

Functional enrichment analysis of Gene Ontology (GO) categories was carried out using standard hypergeometric test [5]. The biological knowledge extraction was complemented through the use of Ingenuity Pathway Analysis (Ingenuity Systems, www.ingenuity.com), which database includes manually curated and fully traceable data derived from literature sources.

SUPPLEMENTAL MATERIALS:

SUPPLEMENTAL MATERIALS:

Antibodies

ANTIBODIES	SUPPLIER	CAT.#	USE
Suz12	Abcam	ab12073	RIP-WB, RNA PULL DOWN
IgG rabbit	Cell Signaling	2729S	RIP & ChIP
Wdr5	Abcam	ab56919	RIP
Suz12	Bethyl	A302-407A	ChIP
H3K27me3	Abcam	ab6002	ChIP
p53 (CM5)	Novocastra	NCL-p53-CM5p	WB&ChIP
p53 (DO-1)	Santa Cruz	sc126	WB&ChIP
Ezh2	Cell Signaling	ac22	WB
Beta-tubulin	Sigma	T5293	WB

Oligonucleotides

Antisense Oligos (ASOs)	sequence	Position
ASO #1	GCCGACTCCCCATCTCTGCC	ex4
ASO #2	GGCACCCATCTCCCTTGCAA	intronic
ASO #3	TAGCTCAGTTCTTCTAACGC	ex3
ASO #4	CCTTTAGTTAGCTTTGGTCT	intronic
ASO CTRL #1	CCTTCCCTGAAGGTTCCTCC	
ASO CTRL #2	TAGTGCGGACCTACCCACGA	

siRNA	sequence	Supplier
siRNA *p53*#1	AGAAGAAAAUUUCCGCAAA	Ambion
siRNA *p53*#2	ACAGCGUGGUGGUACCUUA	Ambion
siRNA control	Not available from supplier	Ambion

	**FORWARD**	**REVERSE**
**shRNA cloning**		
Ezh2	CCGGCGGCTCCTCTAACCATGTTTACT	AATTCAAAAACGGCTCCTCTAACCATG
Scramble	CCGGCAACAGCCACAACGTCTATATCT	AATTCAAAAACAACAGCCACAACGTCT

qRT-PCR and qPCR primers

**GENE**	**FORWARD**	**REVERSE**	**FIGURE**
**mouse qPCR**			
Gapdh	GGGAAATTCAACGGCACAGT	AGATGGTGATGGGCTTCCC	1E, 1F, 1G, 2A, 2C, 3C, 4A,<br>S2B, S2C, S2D, S3A, S3H, S4C,S5F,S5G
Pint A.1	CGCGCACGTATTCTTGTATG	AGGAACCCGAAAGACACCTT	S3B
PintA.2	CGGTGTAGTGTTCAGCCTCA	GGTGGCAGACTCCTGTTAGC	1E, 1F, 1G, 2A, 2C, 3C, 4A,<br>4D, S2C, S2D, S3A,S3H, S4C,S5G
Pint C	GACCAGGTTGCTGCTATTCTTC	AAGGCTGCACAGATACCTGACT	S2B
Pint B	CGGTGTAGTGTTCAGCCTCA	TGGCTCTGATCTGTGGTCA	S2B
Ezh2	CTCTTCTGTCGACGATGTTTTAAG	GGGTGTTGCATGGAAGGA	S5F
U1	GATCACGAAGGTGGTTTTCC	TAAAGGGGAGAGCACAAACG	4A
Arc	GGTGAGCTGAAGCCACAAAT	TTCACTGGTATGAATCACTGCTG	S4C
Gadd45b	CTGCCTCCTGGTCACGAA	TTGCCTCTGCTCTCTTCACA	S4C
Celf5	GGGACCAAGGACAGACCAG	CTGGAGGTATATGGTGTTGACG	S4C
Mmp15	GCCTGCAGCTTCTTTCCTC	CCCTTTGGTCCTAGGTGAGA	S4C
Egr2	CTCCAGGTAGCGAGGGAGTT	CCTTGGCGGTCATCATTT	S4C
Nkx2-9	GTGCGTTCCACAGACTGCT	GAGTCTGCAGGGCTTGTCTC	S4C
Atm	CCCCACCCTGATAAGCAAG	CTCTGGTCCCTCAACACGAT	S4C
Angpt2	GATCAGACCAGTGAAATAAACAAGC	GAGCTCTGCTTGGACACCA	S4C
Amigo2	GCTGTCATCTCCAACAAAGA	CTTGGCTGAATCTGGGATAAG	S4C
Serpina3N	CTTCGCCACAACAACATCAC	CTCTTTACCGACTTCAGCCTATT	S4C
Fas	TGTCAACCATGCCAACCT	CCCTTCTCCCAATTCTCTTCTT	S4C
Il1r1	AGGTGGAGGACTCAGGATATT	CCAGGGTCATTCTCTAACACAG	S4C
Pik3r1	CCAGTCCCTGACTTCAAGAATTA	CCCTGTACCAAAGCACTATGT	S4C
Jag2	GACAATGACACCACTCCAGATG	CATCACAGCGTACTCGGATCT	S4C
Tgfbi	CAGTGGTACCAGAGGAAGA	CAGATTTGAGAGCGGAAGAG	S4C
p53	ACGCTTCTCCGAAGACTGG	AGGGAGCTCGAGGCTGATA	S2B

**ChIP p53 qPCR mouse**	**FORWARD**	**REVERSE**	**FIGURE**
Pint p53RE#1	TCCTCTGGAGTGAGGAGGAA	CCTGTCTCAGAGTCCCCATC	1C
Pint p53RE#2	TGCACTGCTATGAACTTGGTTT	GTGACCCAGCAAGTCATTGG	1C
Pint p53RE#3	CCAGCTCAGCTCTGAGTCAC	GCTTTCACGAGGAGACTGGT	1C
Cdkn1a p53RE	GAGACCAGCAGCAAAATCG	CAGCCCCACCTCTTCAATTC	1C
Nc6 (control-)	GCTCCCTCAGCTTCAACATC	CAGAGTGATGAAAGGGTGGA	1C

ChIP Suz12 qPCR	FORWARD	REVERSE	FIGURE
Gm1337	CCCTCGGGATGAGGACTAA	CCAAACTCACCCCAGTCTTC	5D,5E, S5B, S5C
Hoxc11	TGTCCTGCTACCTCTACGGG	AATGCTTCTGCAAATCCAGG	5D,5E, S5B, S5C
Chd7	TCTGAAACCCAGGCGATTT	GTGCTGAGCTCCATGTGAAA	5D,5E, S5B, S5C
Lrp2	CTCGTGCATGCTCTCACC	AGCGTGGGAGGCAGTTTT	5D,5E, S5B, S5C
Kdr	AAGTCACAGAGGCGGTATGC	AACCTGGCTGACCCGATT	5D,5E, S5B, S5C
Rab20	AAGACTCGGCACTGGGTAAA	AGGCGACTGGGAAGTAGAGTT	5D,5E, S5B, S5C
Chd6	GGCAGGAAAATAAAAAGTTAAGAG AAG	TGAGCTAAACTTCACCAAATAATA GC	5D,5E, S5B, S5C
Frmda	GGTGTGTGTGTTTCGAGCA	AAGGCAGAGACCGTACAGTTG	5D,5E, S5B, S5C
Foxq1	CTCGGCCTAAGCCCTTTTAG	CTGGTAGAGCTGACACTTTTTGG	5D,5E, S5B, S5C
Tll2	GCCAATCACTTATGCGTCCT	GGGTTCCTCCGAAGTTACG	5D,5E, S5B, S5C
Hey1	GGCGCTTCTCGATGATCT	TCATAACGTAAATGCCCTCCTT	5D,5E, S5B, S5C
Rasgrf2	GATGGTATGGAACCACTTCTC	CGATGATGGTAGAACTGACTG	5D,5E, S5B, S5C
Elovl3.F1	GGGCAAACTTGGTTCTTAGG	CCCTCAGCAGTTATCCAATC	5D,5E, S5B, S5C
4930486G11Rik	TGCCTGCTCTCTGTACTC	GCGACCTACTTATCAGGTAAAG	5D,5E, S5B, S5C
Nefm	CAAGCCTTGCGGTGATT	AGGTCTGGAACTCAAGGG	5D,5E, S5B, S5C
Tmem171	AAGGAGCTGGTCCCTTAG	GACTTTCCCTGTTGCTCTTC	5D,5E, S5B, S5C
Gpr84	GTGAGGATGGAGACAGAATTG	CTTTCTCCGTGGACACATAC	5D,5E, S5B, S5C
Hcn1	GCGGCAGTATGGTTTCAT	TTTCCTGCTCCTTCTCCA	5D,5E, S5B, S5C
Sox1	ACAGGAACGGAGACTTCGAG	CCACTTTCTGGGTCTGAAGC	5D,5E, S5B, S5C
Nxph4.F1	GGGGCTTGAGGAGGGTAAG	GACCAGCCACAACCCTTG	5D,5E, S5B, S5C
Bmp3.F1	GCTGCTCTGTCTATGGCTAGG	GTGCAAGTTTGGTCTCTGTCC	5D,5E, S5B, S5C

HUMAN qPCR	FORWARD	REVERSE	FIGURE
hGAPDH	AGCCACATCGCTCAGACAC	GCCCAATACGACCAAATCC	6A, 6B, 6E, S6C, S6D
PINT	GAACGAGGCAAGGAGCTAAA	AGCAAGGCAGAGAAACTCCA	6A, 6B, 6E, S6C, S6D

ChIP p53 qPCR human	FORWARD	REVERSE	FIGURE
PINT p53RE#1	TTTAGCTCCTTGCCTCGTTC	TCTACGTGCGCATCATTTTC	6C
PINT p53RE#2	TGGGTCACTGACTAGGGAGAA	GGGTCTAGGGTGGAAGGAAG	6C
PINT p53RE#3	CCTCGAGATGACACGCAGT	GAGGGTGCATGGATCATAGG	6C
PERP p53RE	GCATGTTCACTCATACTAGTTTTGCA	GAAAATATCCTCCTGATGTATTCT TTCTTA	6C
NC6 (control-)	GCTCCCTCAGCTTCAACATC	CAGAGTGATGAAAGGGTGGA	6C

A

B

C

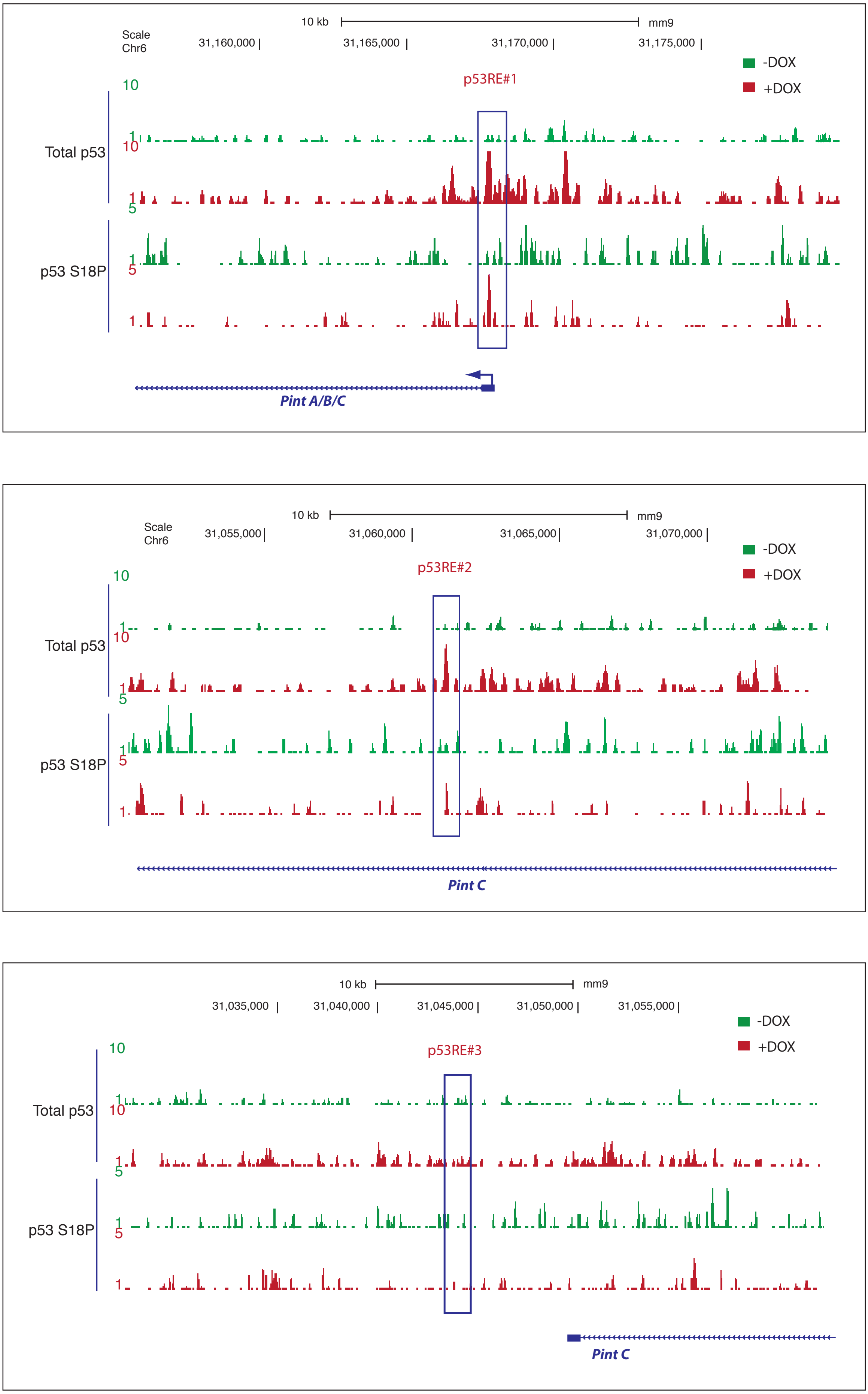
10 kb
mm9
Scale
Chr6
31,160,000
31,165,000
31,170,000
31,175,000
-DOX
+DOX
p53RE#1
Total p53
p53 S18P
Pint A/B/C
31,055,000
31,060,000
31,065,000
31,070,000
p53RE#2
Pint C
31,035,000
31,040,000
31,045,000
31,050,000
31,055,000
p53RE#3

Figure 1. p53 ChIP-seq data on *PINT* region of mESCs.

Total p53 and phosphorylated p53 (p53 S18P) ChIP-seq signal from mESCs treated with doxorubicin (red tracks) or untreated (green tracks) in the regions corresponding to *PINT p53RE#1* (A), *p53RE#2* (B) and *p53RE#3* (C). Data were downloaded from [7].

A

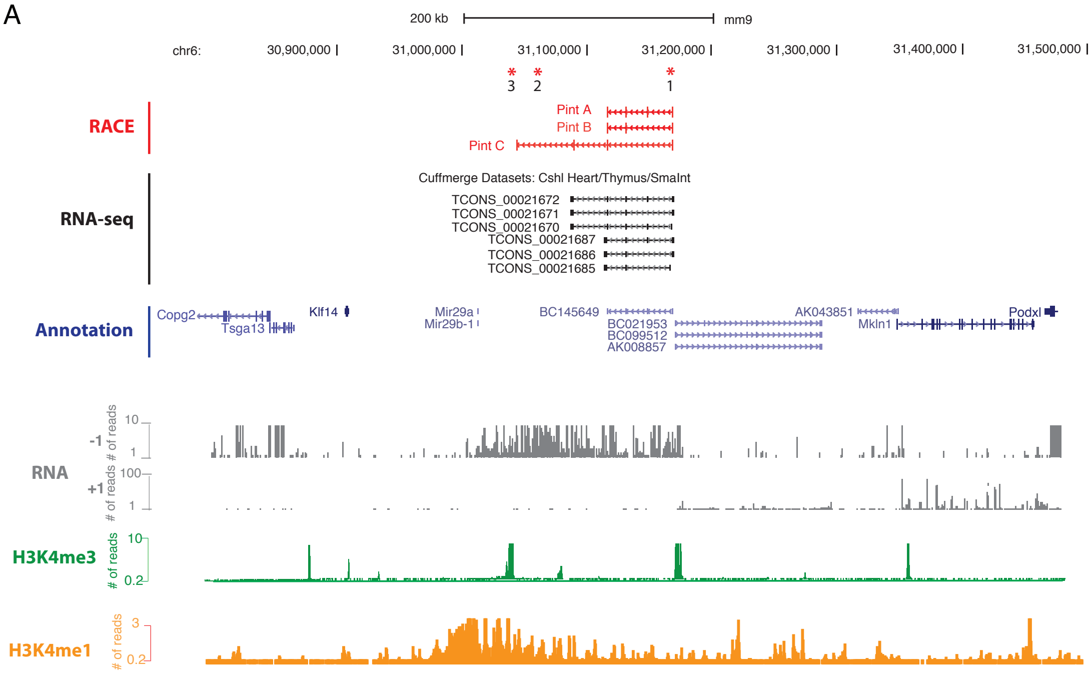

B

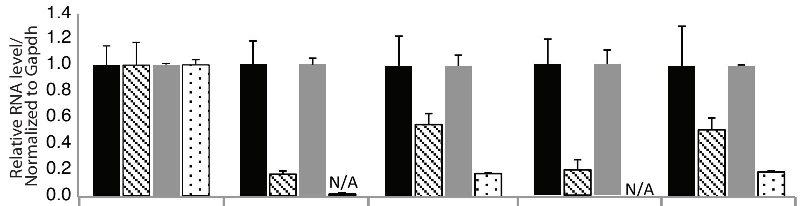

	Gapdh	p53	*Pint A*	*Pint B*	*Pint C*
siRNA control	17.34	21.59	24.05	37.06	25.38
siRNA p53	17.26	23.99	24.69	39	26.31
p53+/+	16.65	20.52	21.64	38.17	22.84
p53-/-	16.7	Undetermined	24.12	Undetermined	25.22

C

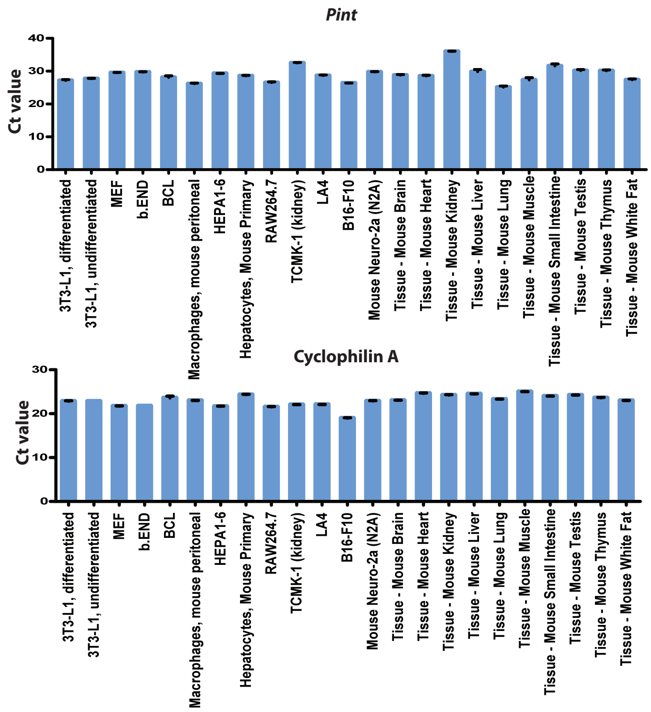

D

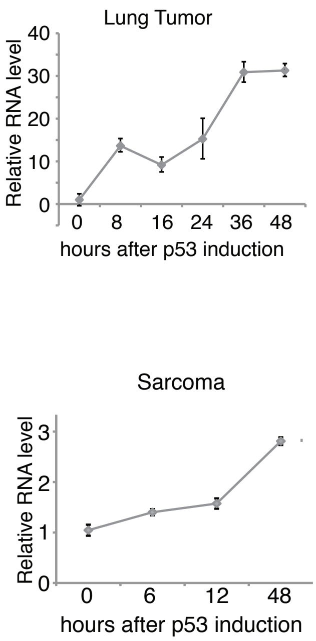

Figure S2. *Pint* is a ubiquitously expressed lincRNA.

(A) Top: Representation of *Pint* isoforms cloned by RACE (red), isoforms determined by Cufflinks analysis of RNA-seq data from heart, thymus and small intestine (black) and annotated transcripts (blue). Bottom: RNA-seq reads form ovary and H3K4me3 and H3K4me1 ChIP-seq (MEFs) data of *Pint* genomic region. Red asterisks indicate the positions of *Pint* p53REs. Data are downloaded from UCSC genome browser [6].

(B) Relative RNA levels of Gapdh, p53 and *Pint* isoforms in MEFs transfected with siRNA control, p53 siRNA and in p53+/+ MEFs or p53-/-MEFs. The graph represents the RNA levels relative to gapdh and the table shows the qRT-PCR Ct values.

(C) *Pint* A (top) and housekeeping gene *cyclophilin A* (bottom) expression levels determined by qRT-PCR of 5ng of total RNA from different mouse cell lines and tissues. –RT controls were performed and no meaningful signal was detected.

(D) *Pint* levels at different times after p53 restoration in lung tumor (top) and sarcoma cell lines (bottom).

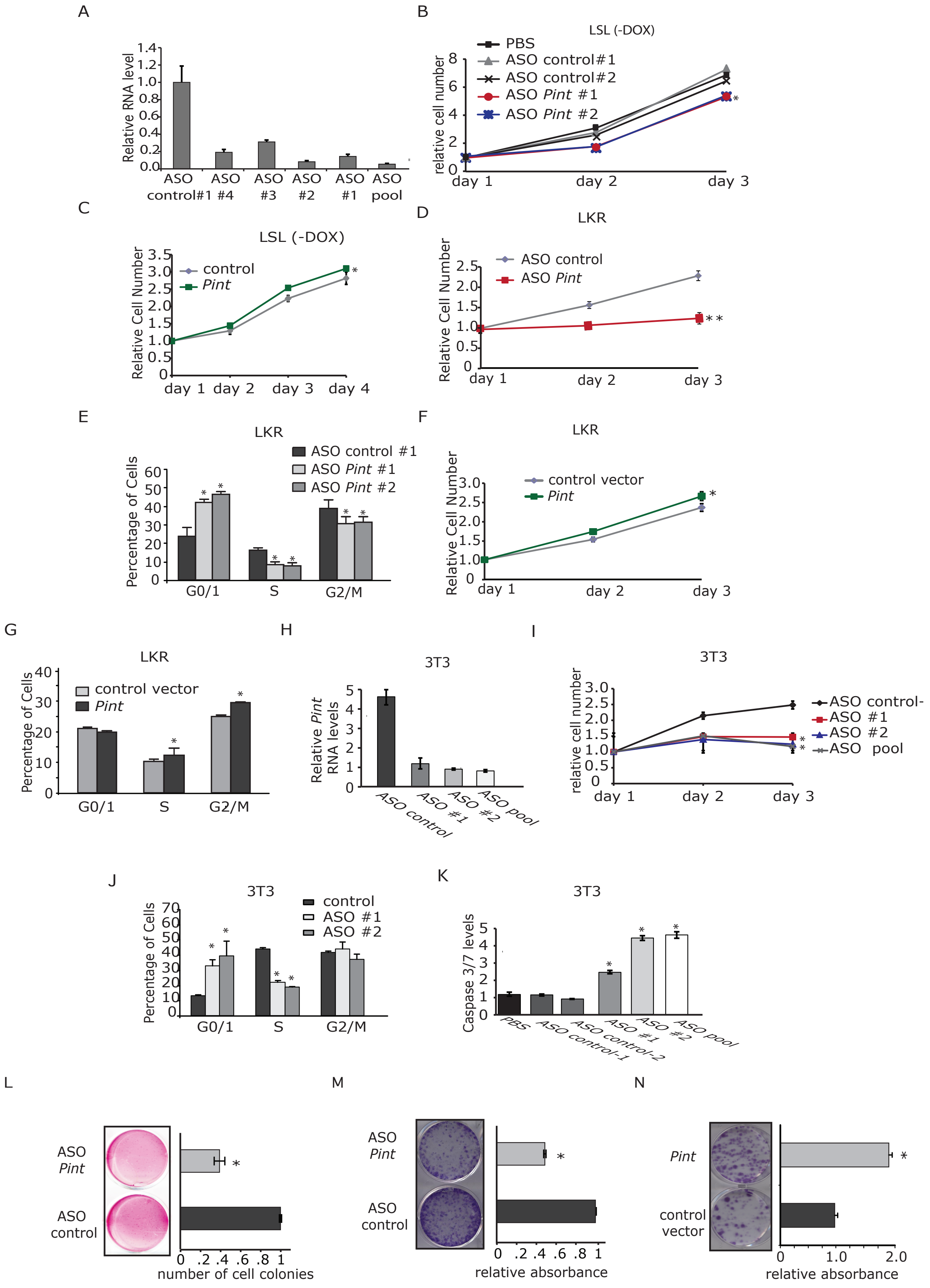
A
Relative RNA level
ASO control#1
ASO #4
ASO #3
ASO #2
ASO #1
ASO pool
B
LSL (-DOX)
PBS
ASO control#1
ASO control#2
ASO Pint #1
ASO Pint #2
relative cell number
day 1
day 2
day 3
C
LSL (-DOX)
control
Pint
Relative Cell Number
day 1 day 2 day 3 day 4
D
LKR
ASO control
ASO Pint
Relative Cell Number
day 1 day 2 day 3
E
LKR
ASO control #1
ASO Pint #1
ASO Pint #2
Percentage of Cells
G0/1
S
G2/M
F
LKR
control vector
Pint
Relative Cell Number
day 1 day 2 day 3
G
LKR
control vector
Pint
Percentage of Cells
G0/1 S G2/M
H
3T3
Relative Pint RNA levels
ASO control
ASO #1
ASO #2
ASO pool
I
3T3
relative cell number
ASO control-
ASO #1
ASO #2
ASO pool
day 1 day 2 day 3
J
3T3
control
ASO #1
ASO #2
Percentage of Cells
G0/1 S G2/M
K
3T3
Caspase 3/7 levels
PBS
ASO control-1
ASO control-2
ASO #1
ASO #2
ASO pool
L
ASO Pint
ASO control
number of cell colonies
M
ASO Pint
ASO control
relative absorbance
N
Pint
control vector
relative absorbance

Figure S3. *Pint* modulates cell proliferation and apoptosis.

(A) Relative *Pint* RNA level of p53-restored $p53^{LSL/LSL}$ MEFs transfected with the indicated ASOs. *Pint* RNA is normalized to Gapdh and represented relative to the condition ASO control#1.

(B) Relative number of p53-restored $p53^{LSL/LSL}$ MEFs transfected with ASOs for *Pint* inhibition or control ASO.

(C) Relative number of p53-restored $p53^{LSL/LSL}$ MEFs cells stably transduced with a retroviral vector expressing *Pint* or empty vector as control.

(D) Relative number of LKR cells transfected with a pool of *Pint* targeting ASOs or control ASO. Cells are treated with 150nM Dox from 24h post transfection.

(E) Percentage of LKR cells in each phase of cell cycle after transfection of two independent *Pint*-targeting ASOs or a control ASO. Cells are treated with 150nM DOX from 24h post transfection for 12h.

(F) Relative number of LKR cells transfected with a plasmid expressing *Pint* or an empty plamid. Cells are treated with 150nM Dox from 24h post transfection.

(G) Percentage of LKR cells in each phase of cell cycle after transfection of a plasmid expressing *Pint* or an empty plasmid. Cells are treated with 150nM Dox from 24h post transfection for 12h.

(H) *Pint* relative levels after transfection of the indicated ASOs in 3T3 MEF cells.

(I) Relative number of 3T3 MEFs transfected with ASOs for *Pint* inhibition or control ASO and treated with 150nM DOX.

(J) Percentage of 3T3 MEFs in each phase of the cell cycle after transfection with the indicated ASOs followed with treatment with DOX for 12h.

(K) Apoptosis levels determined by caspase-3 levels in 3T3 MEFs treated as in (J)

(L) Growth independent of attachment assay of 3T3 MEFs treated with a pool of *Pint*-targeting ASOs or a control ASO.

(M) Growth independent of contact assay of cells treated like in (L).

(N) Growth independent of contact assay of 3T3 MEFs transfected with a plasmid expressing *Pint* or an empty plasmid.

Values are average of three biological replicates +/-STD. Asterisks represent statistical significance (*P<.05, **P<.01, ***P<.0001).

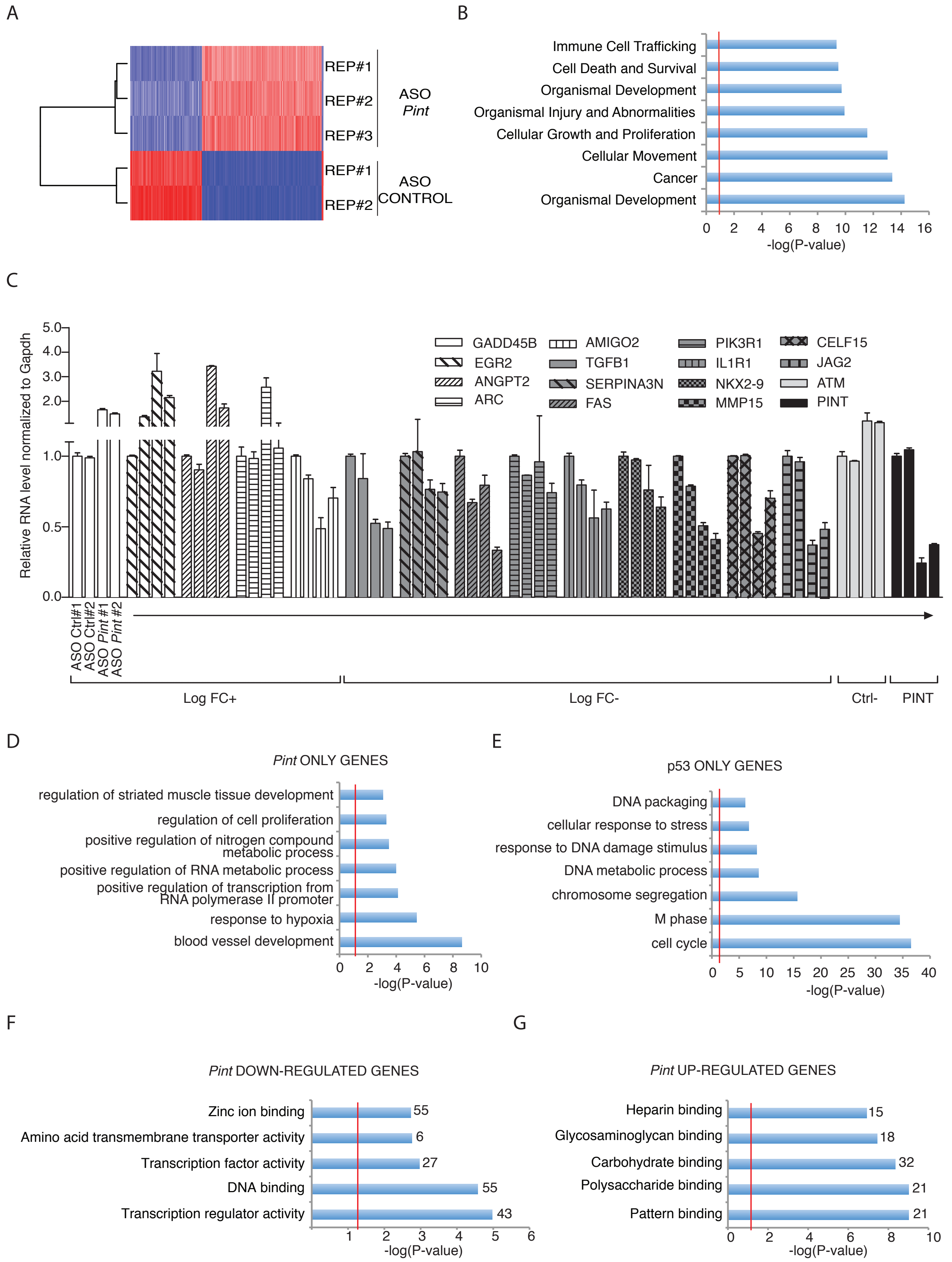
A
REP#1
REP#2
REP#3
ASO
Pint
REP#1
REP#2
ASO
CONTROL
B
Immune Cell Trafficking
Cell Death and Survival
Organismal Development
Organismal Injury and Abnormalities
Cellular Growth and Proliferation
Cellular Movement
Cancer
Organismal Development
0 2 4 6 8 10 12 14 16
-log(P-value)
C
Relative RNA level normalized to Gapdh
5.0
4.0
3.0
2.0
1.0
0.5
0.0
GADD45B
EGR2
ANGPT2
ARC
AMIGO2
TGFB1
SERPINA3N
FAS
PIK3R1
IL1R1
NKX2-9
MMP15
CELF15
JAG2
ATM
PINT
ASO Ctrl#1
ASO Ctrl#2
ASO Pint #1
ASO Pint #2
Log FC+
Log FC-
Ctrl-
PINT
D
Pint ONLY GENES
regulation of striated muscle tissue development
regulation of cell proliferation
positive regulation of nitrogen compound metabolic process
positive regulation of RNA metabolic process
positive regulation of transcription from RNA polymerase II promoter
response to hypoxia
blood vessel development
0 2 4 6 8 10
-log(P-value)
E
p53 ONLY GENES
DNA packaging
cellular response to stress
response to DNA damage stimulus
DNA metabolic process
chromosome segregation
M phase
cell cycle
0 5 10 15 20 25 30 35 40
-log(P-value)
F
Pint DOWN-REGULATED GENES
Zinc ion binding 55
Amino acid transmembrane transporter activity 6
Transcription factor activity 27
DNA binding 55
Transcription regulator activity 43
1 2 3 4 5 6
-log(P-value)
G
Pint UP-REGULATED GENES
Heparin binding 15
Glycosaminoglycan binding 18
Carbohydrate binding 32
Polysaccharide binding 21
Pattern binding 21
0 2 4 6 8 10
-log(P-value)

Figure S4. *Pint* regulates the expression on of genes involved in proliferation and survival.

(A) Genes affected by *Pint* inhibition in p53-restored DOX-treated $p53^{LSL/LSL}$ MEFs (B>3). Colors represent transcripts above (blue) or below (red) the global median scaled to 2-fold activation or repression, respectively.

(B) Significant biofunctions of genes affected by *Pint* inhibition (B>3). The red line indicates P=0.05.

(C) Validation by qRT-PCR of expression of genes upon depletion of *Pint* with two independent ASOs (ASO *Pint*#1 and ASO *Pint*#2) compared to two independent control ASOs (ASO Ctrl#1 and ASO Ctrl#2). For each mRNA, values are normalized by Gapdh and represented relatively to the condition ASO ctrl#1. Values are the average of 4 replicates +/-STD.

(D) Biofunctions of genes regulated by *Pint* but not p53.

(E) Biofunctions of genes regulated by p53 but not *Pint*.

(F) Biofunctions of genes downregulated by *Pint*.

(G) Biofunctions of genes upregulated by *Pint*.

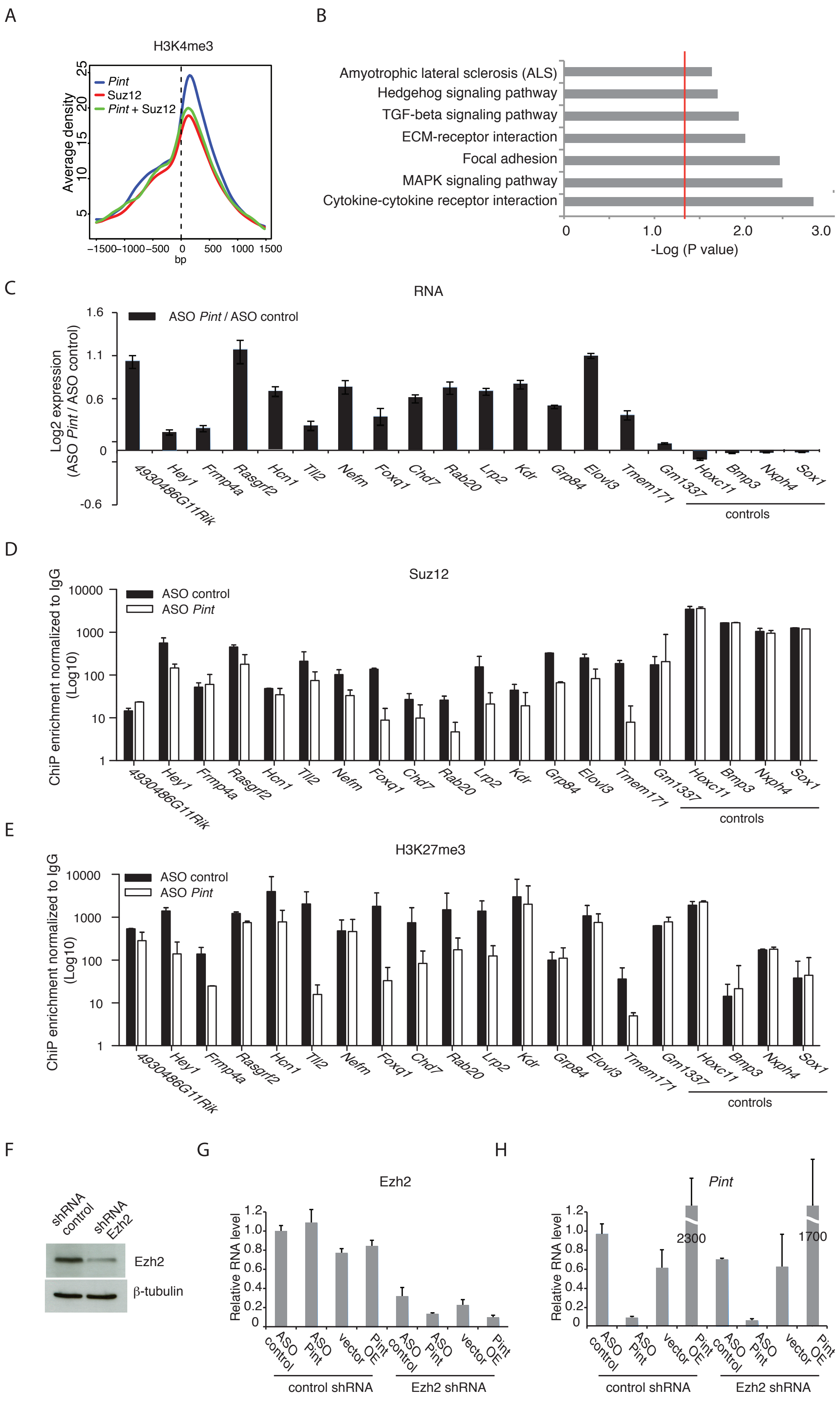
A
H3K4me3
Average density
Pint
Suz12
Pint + Suz12
bp
B
Amyotrophic lateral sclerosis (ALS)
Hedgehog signaling pathway
TGF-beta signaling pathway
ECM-receptor interaction
Focal adhesion
MAPK signaling pathway
Cytokine-cytokine receptor interaction
-Log (P value)
C
RNA
ASO Pint / ASO control
Log2 expression (ASO Pint / ASO control)
controls
D
Suz12
ASO control
ASO Pint
ChiP enrichment normalized to IgG (Log10)
controls
E
H3K27me3
ASO control
ASO Pint
ChiP enrichment normalized to IgG (Log10)
controls
F
shRNA control
shRNA Ezh2
Ezh2
β-tubulin
G
Ezh2
Relative RNA level
control shRNA
Ezh2 shRNA
H
Pint
Relative RNA level
2300
1700
control shRNA
Ezh2 shRNA

Figure S5. PRC2 is required for *Pint* function.

(A) Average H3K4me3 ChIP-seq signal around the TSS of genes regulated by *Pint* but not bound by Suz12 (blue), genes bound by Suz12 but not regulated by *Pint* (red) and genes regulated by *Pint* and bound by Suz12 (green) in mESCs [9].

(B) Top significant KEGG pathways of genes regulated by *Pint* and bound by Suz12.

(C) Relative mRNA level of H3K27me3-regulated genes in DOX-treated p53-reconstituted $p53^{LSL/LSL}$ MEFs transfected with *Pint*-ASOs. Values are average of three replicates +/-STD.

(D) and (E) Relative Suz12 (D) or H3K27me3 (E) enrichment in promoter regions of H3K27me3-regulated genes [32] in DOX-treated p53-reconstituted $p53^{LSL/LSL}$ MEFs treated with *Pint*-ASOs or control ASOs determined by ChIP-qPCR. Enrichment values are relative to IgG, and are the average of three biological replicates +/-STD.

(F) Ezh2 protein levels in Ezh2 shRNA and shRNA control stable cell lines. Beta-tubulin levels are shown as loading control.

(G) and (H). Relative Ezh2 (G) and *Pint* (H) RNA levels in the shRNA control stable cell line (left) and Ezh2 shRNA cell line (right) treated as indicated. Values are the average of four replicates +/- STD.

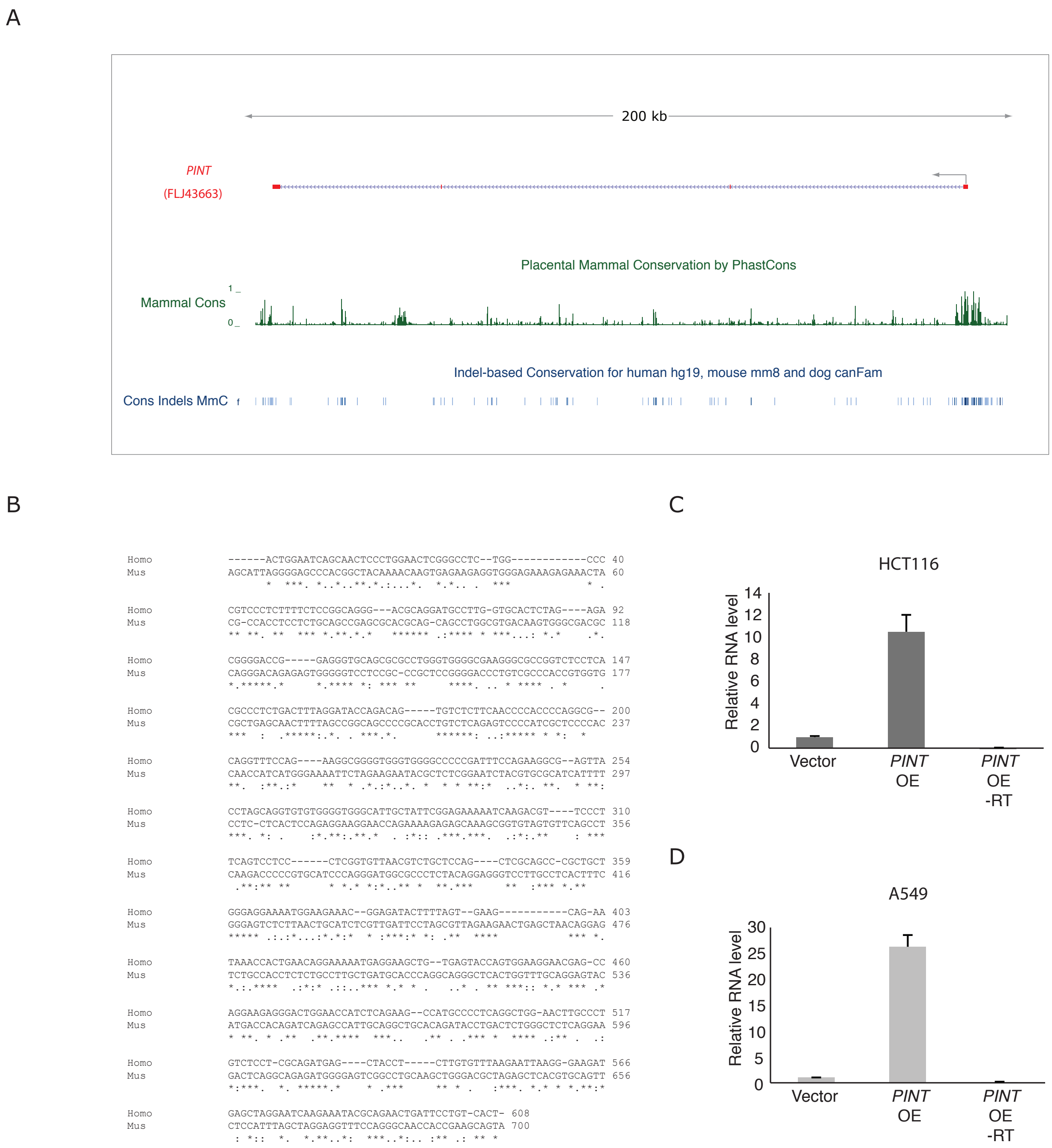

Figure S6. *Pint* human ortholog.

(A) Conservation across placental mammalian species determined by PhasCons (green) and indel-based conservation for human, mouse and dog determined by canFam (blue) in *PINT* genomic locus. Data were obtained from UCSC genome browser [6].

(B) Alignment of mouse (*Pint* A) and human *PINT* (FLJ43663) 5' sequences.

(C) and (D) Relative *PINT* levels in HCT116 (C) or A549 (D) cell stable cells lines. Values are normalized by *GAPDH*.

SUPPLEMENTAL TABLE LEGENDS:

Additional file 2, Table S1. Genomic coordinates and sequences of p53REs found in mouse and human *PINT* genomic loci.

Additional file 3, Table S2. Genes affected by *PINT* inhibition by ASO transfection of p53-reconstituted $p53^{LSL/LSL}$ MEFs treated with DOX (B>3).

Additional file 4, Table S3. Predicted upstream regulators of genes affected by *Pint* knockdown.

Additional file 5, Table S4. Genes affected by p53 inhibition by siRNA transfection of p53-reconstituted $p53^{LSL/LSL}$ MEFs treated with DOX (B>3).

Additional file 6, Table S5. Genes commonly affected by *Pint* and p53 inhibitions.

Additional file 7, Table S6. Genes regulated by *Pint* (B>3) found to be bound by Suz12 in mESC [8].

Additional file 8, Table S7. Human samples included in this study

Pint SEQUENCES:

- **mus musculus *Pint* A**
-

```
CCCACGGCTACAAACCAAGTGAGAAGAGGTGGGAGAAAGAGAAACTAGCCACCTCCTCTGCA
GCCGAGCGCACGCAGCAGCCTGGCGTGACAAGTGGGCGACGCCAGGGACAGAGAGTGGGGGT
CCTCCGCCCGCTCCGGGGACCCTGTCGCCCACCGTGGTGCGCTGAGCAACTTTTAGCCGGCA
GCCCCGCACCTGTCTCAGAGTCCCCATCGCTCCCCACCAACCATCATGGGAAAATTCTAGAA
GAATACGCTCTCGGAATCTACGTGCGCATCATTTTCCTCCTCACTCCAGAGGAAGGAACCAG
AAAAGAGAGCAAAGCGGTGTAGTGTTCAGCCTCAAGAACCCCCGTGCATCCCAGGGATGGCG
CCCTCTACAGGAGGGTCCTTGCCTCACTTTCGGGAGTCTCTTAACTGCATCTCGTTGATTCC
TAGCGTTAGAAGAACTGAGCTAACAGGAGTCTGCCACCTCTCTGCCTTGCTGATGCACCCAG
GCAGGGCTCACTGGTTTGCAGGAGTACATGACCACAGATCAGAGCCATTGGCTGCACAGATA
CCTGACTCTGGGCTCTCAGGAAGACTCAGGCAGAGATGGGGAGTCGGCCTGCAAGCTGGGAC
GCTAGAGCTCACGTGCAGTTCTCCATTTAGCTAGGAGGTTTCCAGGGCAACCACCGAAGCAG
TAATTAAAGATGAAGAGCTAAAAGGTAAGTACTTCCAAACCTGAATCCTGAAGGAGGTGGTC
CGCAGGCTGGTGTCACAGTGTGCACTCTTATTCACTTAACATTAAGTAAATAATTTTTTAAA
TACATTTTTTGAAAACCTCTGATGAGAGGATACACACTAAATTTAGGATGTTCCATGCTCTG
CCTTTGATTTGTATCTTTTTTCACGCGCGCACGTATTCTTGTATGTACGTACACACCACACA
CACACACACATTTTTATGCTTCCACCAGCCAGTCATGGGACCAGGCATTGGGATACCAGGGC
TTAAGGTGTCTTTCGGGTTCCTGTTTTGAACGTCCTAGCATGCATGTATATATATATACATA
CATACATATATATATATACACATTTAAGGCATGCAAACGAGCAACTGCAGAGGCGTCTACAG
TACTTAGCGTGTAGCAGCTTCATCCAGGCTCTCCAGGCAGT
```

> mus musculus *Pint* B

GCAGCAGCCTGGCGTGACAAGTGGGCGACGCCAGGGACAGAGAGTGGGGGTCCTCCGCCCGC
TCCGGGGACCCTGTCGCCCACCGTGGTGCGCTGAGCAACTTTTAGCCGGCAGCCCCGCACCT
GTCTCAGAGTCCCCATCGCTCCCCACCAACCATCATGGGAAAATTCTAGAAGAATACGCTCT
CGGAATCTACGTGCGCATCATTTTCCTCCTCACTCCAGAGGAAGGAACCAGAAAAGAGAGCA
AAGCGGTGTAGTGTTCAGCCTCAAGGCATCTCGTTGATTCCTAGCGTTAGAAGAACTGAGCT
AACAGGAGTCTGCCACCTCTCTGCCTTGCTGATGCACCCAGGCAGGGCTCACTGGTTTGCAG
GAGTACATGACCACAGATCAGAGCCATTGCAGGCTGCACAGATACCTGACTCTGGGCTCTCA
GGAAGACTCAGGCAGAGATGGGGAGTCGGCCTGCAAGCTGGGACGCTAGAGCTCACGTGCAG
TTCTCCATTTAGCTAGGAGG

- **mus musculus *Pint* C**

AATGATGGACATGATATAATGAAACAACATTGTGGAGAGGAAAGCATTAGGGGAGCCCACGG
CTACAAAACAAGTGAGAAGAGGTGGGAGAAAGAGAAACTACGCCACCTCCTCTGCAGCCGAG
CGCACGCAGCAGCCTGGCGTGACAAGTGGGCGACGCCAGGGACAGAGAGTGGGGGTCCTCCG
CCCGCTCCGGGGACCCTGTCGCCCACCGTGGTGCGCTGAGCAACTTTTAGCCGGCAGCCCCG
CACCTGTCTCAGAGTCCCCATCGCTCCCCACCAACCATCATGGGAAAATTCTAGAAGAATAC
GCTCTCGGAATCTACGTGCGCATCATTTTCCTCCTCACTCCAGAGGAAGGAACCAGAAAAGA
GAGCAAAGCGGTGTAGTGTTCAGCCTCAAGGCTGCACAGATACCTGACTCTGGGCTCTCAGG
AAGACTCAGGCAGAGATGGGGAGTCGGCCTGCAAGCTGGGACGCTAGAGCTCACGTGCAGTT
CTCCATTTAGCTAGGAGGTTTCCAGGGCAACCACCGAAGCAGTAATTAAAGATGAAGAGCTA
AAAGAGAGAAGAATAGCAGCAACCTGGTCTCTTTTCACGGAACACAGTAAGCCACCAAAGAG
GTGTGGACAGCCAGCGACCTCCACGGAAATCATAAGGGC

- **Homo sapiens *PINT* (BC130416)**
-

GCTTGAAAGCCGTGGTGATGGTAATTATGTATCAAATGCCTGGTTCTATTTCTGTTATTATT
GTTTTGTCATTTCTGTTTTCCCAGCGATCTGACTGAACTCGCAGAGGGACAAATCCAGTTTT
TCTTTTTGACTTTTGTCAAACTAAATCAGGCCTGATAGAAAACTCATTGCTCTCCGGGGAAA
CAAAGTAGGAGCCACGAAATGTCATTTTAACAGAGCGTGGGTTTGGTGACTGTAGGAAAGGA
TTTGAGGACGCTCCTTCTGTTCGGCTTCCTATGTCATGAGCACAGGCTCCACGCACGCACAG
ACACCACGGCTCCCGGATGCTGTGGCTCCCCGATCGGGGCTCCTGCAGCGCCAGAAGCCCCT
CCGGGATGCTTCGAGGGGCTCCCGGTGGGTGGAGGTACGGACGCCGCTGCGGCCGCCGCCGC
CAGTCCTGCTGCTGTTGTTGCTGCTGCAGTCACGTGGGAGCCCCTTTAAGTTTCCATAGAGA
GGCCTCTCTGGTGTCACATGATGGACATGATATAATGAAACAACATTGTGGAGAGGAAAGCA
TTAGGGGAGCCCACGGCTACAAAAACAAGTGAGTGAGAAGAGGTGGGAGGAAGAGAAACTAC
GCCACCTCCCCTGCAGCCGAGTGCACGCAGCAGCCTGGCGTGACAAGTGGGCGACGCCGGGG
GGCAGGGAGCCGGGGTCCTTGGCCCTGGCCGGGGACCCCACCGCCCACCGCGCGGAGGACAA
CTTTTAGCCGGCAGCCCAGACCAGCGCGGCACCTGTCTCCGGAGTCTCCACCGCTCCTCCCG
ATTCATCCCAGGGAAATTCTCAAGAATACGCTCTACAAATCTACGTGCGCATCATTTTCACC
TCGCGTCGCGCCCGGGAGGAAGGAACGAGGCAAGGAGCTAAAGCAGCGTGCGTTCAGCCCTG
GGGCATTTTATTAATGCTTTTACGAGTTAGAAGAGTTGGGATAATTTGCCATCTGGAGTTTC
TCTGCCTTGCTGATCTGAGCTCAGACCTGCCAATTTACCAGAGATAATTGATAACACCCTCT
AACAGCTGAGAGGAAAATGGAAGAAACGGAGATACTTTTAGTGAAGCAGAATAAACCACTGA
ACAGGAAAAATGAGGAAGCTGTGAGTACCAGTGGAAGGAACGAGCCAGGAAGAGGGA

SUPPLEMENTARY REFERENCES

1. Irizarry, RA., Bolstad, BM., Collin, F., Cope LM., Hobbs, B., Speed, TP. **Summaries of affymetrix genechip probe level data**. Nucleic Acids Res 2003. **31**(4), e15.

2. Gentleman, V. Carey, S. Dudoit, R. Irizarry, and W. Huber (ed.), **Bioinformatics and computational biology solutions using R and Bioconductor**. Springer, New York, NY.

3. Smyth, G. K. **Linear models and empirical Bayes methods for assessing differential expression in microarray experiments.** Statistical Applications in Genetics and Molecular Biology 2004. **3**, No. 1, Article 3.

4. Perez-Llamas, C., and Lopez-Bigas, N. **Gitools: analysis and visualisation of genomic data using interactive heat-maps.** PLoS One 2001. **6**, e19541.

5. Draghici, S. **Data analysis tools for DNA microarrays. Chapman Hall/CRC, London.** 2003.

6. Kent WJ, Sugnet CW, Furey TS, Roskin KM, Pringle TH, Zahler AM, Haussler D: **The human genome browser at UCSC**. *Genome Res* 2002, **12**(6):996-1006.

7. Li M, He Y, Dubois W, Wu X, Shi J, Huang J: **Distinct regulatory mechanisms and functions for p53-activated and p53-repressed DNA damage response genes in embryonic stem cells**. *Mol Cell* 2012, **46**(1):30-42.

8. Ku M, Koche RP, Rheinbay E, Mendenhall EM, Endoh M, Mikkelsen TS, Presser A, Nusbaum C, Xie X, Chi AS, Adli M, Kasif S, Ptaszek LM, Cowan CA, Lander ES, Koseki H, Bernstein BE: **Genomewide analysis of PRC1 and PRC2 occupancy identifies two classes of bivalent domains**. *PLoS Genet* 2008, **4**, e1000242.

9. Mikkelsen TS, Ku M, Jaffe DB, Issac B, Lieberman E, Giannoukos G, Alvarez P, Brockman W, Kim TK, Koche RP, Lee W, Mendenhall E, O'Donovan A, Presser A, Russ C,

Xie X, Meissner A, Wernig M, Jaenisch R, Nusbaum C, Lander ES, Bernstein BE: **Genome-wide maps of chromatin state in pluripotent and lineage-committed cells**. *Nature* 2007, **448**(7153):553-560
